# A σ^B^-dependent sRNA provides a repressor function to the general stress response in *Clostridioides difficile*

**DOI:** 10.1128/mbio.01923-26

**Published:** 2026-10-26

**Authors:** Johannes Sulzer, Manuela Fuchs, Kassy Donohoe, Nile U. Bayard, Christopher Birk, Vanessa Lamm-Schmidt, Joseph P. Zackular, Franziska Faber

**Affiliations:** 1 University of Würzburg, Medical Faculty, Institute for Hygiene and Microbiology 9190, Würzburg, Germany; 2 Helmholtz Centre for Infection Research, Helmholtz Institute for RNA‐based Infection Research 557383, Würzburg, Germany; 3 Department of Pathology and Laboratory Medicine, Perelman School of Medicine, University of Pennsylvania 6572, Philadelphia, Pennsylvania, USA; 4 Division of Protective Immunity, Children’s Hospital of Philadelphia 6567, Philadelphia, Pennsylvania, USA; 5 The Center for Microbial Medicine, Children’s Hospital of Philadelphia 6567, Philadelphia, Pennsylvania, USA; 6 Cluster for Nucleic Acid Sciences and Technologies–NUCLEATE, Bavaria, Germany; The Pennsylvania State University, University Park, Pennsylvania, USA

**Keywords:** sigma B, stress response, *C. difficile*, Wood-Ljungdahl pathway, sRNA, small regulatory RNA, Hfq

## Abstract

*Clostridioides difficile* is an anaerobic, gram-positive gastrointestinal pathogen that poses a significant health threat worldwide. A recent RIL-seq characterization of the Hfq-mediated RNA-RNA interactome revealed that small regulatory RNAs (sRNAs) are key post-transcriptional regulators of virulence-related processes in *C. difficile*, including the initiation of sporulation. In the model Proteobacteria species *E. coli* and *S. enterica*, sRNAs often function as non-coding elements in vital stress responses mediated by alternative sigma factors, acting as repressors within these regulatory networks. However, whether this principle is broadly conserved across bacterial phyla remains largely unexplored. Here, we demonstrate that the Hfq-dependent sRNA SrsA is part of the regulatory network of the alternative sigma factor σ^B^, which governs the general stress response in Firmicutes. Consequently, SrsA is highly expressed during the transition from exponential to stationary phase and under infection-related stress conditions, such as oxidative stress. We show that SrsA represses a highly conserved small protein family that regulates sporulation and three genes in the methyl-fixing branch of the Wood-Ljungdahl pathway (WLP). Infection of mice further revealed that SrsA contributes to disease severity and bacterial recovery from antibiotic treatment in an acute and relapse mouse model of *C. difficile* infection. In summary, our data suggest that integrating SrsA into the σ^B^ regulon helps coordinate sporulation and vital metabolic pathways of *C. difficile* in response to infection-relevant stress conditions.

**IMPORTANCE:**

The central role of sRNAs as non-coding arms of stress responses represents a paradigm of sRNA function in Gammaproteobacteria. However, it remains unclear how broadly this regulatory principle is conserved across the bacterial tree of life. In our study, we identified the sRNA SrsA as a non-coding regulator involved in the sigma B-mediated stress response to nutrient limitation and radical stress in the intestinal pathogen *Clostridioides difficile*. We demonstrate that SrsA suppresses the production of a conserved family of spore regulators and of enzymes of the Wood-Ljungdahl pathway, establishing a role in regulating sporulation and gut colonization. Our work provides novel insights into the regulation of the Wood-Ljungdahl pathway, which is usually associated with autotrophic bacteria, and highlights the metabolic flexibility this anaerobic gut pathogen has evolved to survive in a nutrient-limited and inflamed gut environment.

## INTRODUCTION

Bacteria live in constantly changing environments that impose diverse stress conditions. This requires a rapid, targeted adaptation of cellular physiology through changes in gene expression. A common strategy is to replace the housekeeping sigma factor σ^A^ with an alternative sigma factor that redirects RNA polymerase to specific gene sets, driving expression of cellular functions that help bacteria adapt to changing environments and stress conditions. Processes regulated by alternative sigma factors in *Clostridioides difficile* include the transition from exponential to the stationary phase of growth by σ^H^ (1); the coordination of the sporulation cascade by σ^E^, σ^F^, σ^G^, and σ^K^ (2); and general stress responses mediated by σ^B^ (3). While sigma factors exclusively activate gene expression, rapid cellular adaptation often requires the downregulation of specific transcripts. In many cases, this is achieved through small regulatory RNAs (sRNAs) encoded within the sigma factor regulon, which act as translational repressors of preexisting mRNAs. Intensive research over the last two decades has uncovered various sRNAs that provide such non-coding repressor functions to stress responses (4–9) in gram-negative bacteria. Much less is known about gram-positive bacteria, which differ in various physiological aspects, including RNA processing (10). To date, multiple σ^B^-dependent sRNAs have been identified in *Staphylococcus aureus* (RsaA, RsaF, and SbrC) and *Listeria monocytogenes* (SbrA and SbrE), respectively (11–16), but RsaA from *S. aureus* remains the only sRNA with experimentally validated targets (11, 12).

Recently, the obligate anaerobic human pathogen *C. difficile* has been established as a gram-positive model organism for RNA biology. As in gram-negative model organisms, *C. difficile* Hfq serves as a global RNA-binding protein (RBP) and sRNA stability factor (17–20). Several Hfq-dependent sRNAs display promoter signatures of specialized sigma factors, including the alternative sigma factor σ^B^, which regulates general stress responses in many Firmicutes (21, 22). In *C. difficile*, the σ^B^ regulon comprises at least 132 target genes (23), and the loss of *sigB* results in the differential expression of approximately 25% of genes at the onset of the stationary phase (3). The viability of a *sigB* mutant is reduced in late stationary phase and in various stress conditions, including acidic pH, oxygen, and other radical stresses, while sporulation is significantly increased. Consistent with its increased sensitivity to various stressors, survival of a *C. difficile sigB* mutant in a mouse infection model was significantly impaired, highlighting the importance of this sigma factor for gut colonization (3).

Here, we describe a regulatory circuit consisting of the stress-response sigma factor σ^B^ and a σ^B^-dependent sRNA, which we have named SrsA (stress-response sRNA A, previously CDIF630nc_028). We demonstrate that SrsA confers a repressor function to the σ^B^ regulon by targeting the translation initiation regions of several mRNAs, thereby acting as a negative post-transcriptional regulator of several enzymes of the Wood-Ljungdahl pathway (WLP) and a group of paralogous proteins that modulate sporulation frequency. Overall, our data suggest that SrsA may downregulate energy-consuming processes to facilitate an optimal σ^B^-induced stress response.

## RESULTS

### SrsA is part of the σ^B^ stress response regulon

**Fig 1:**
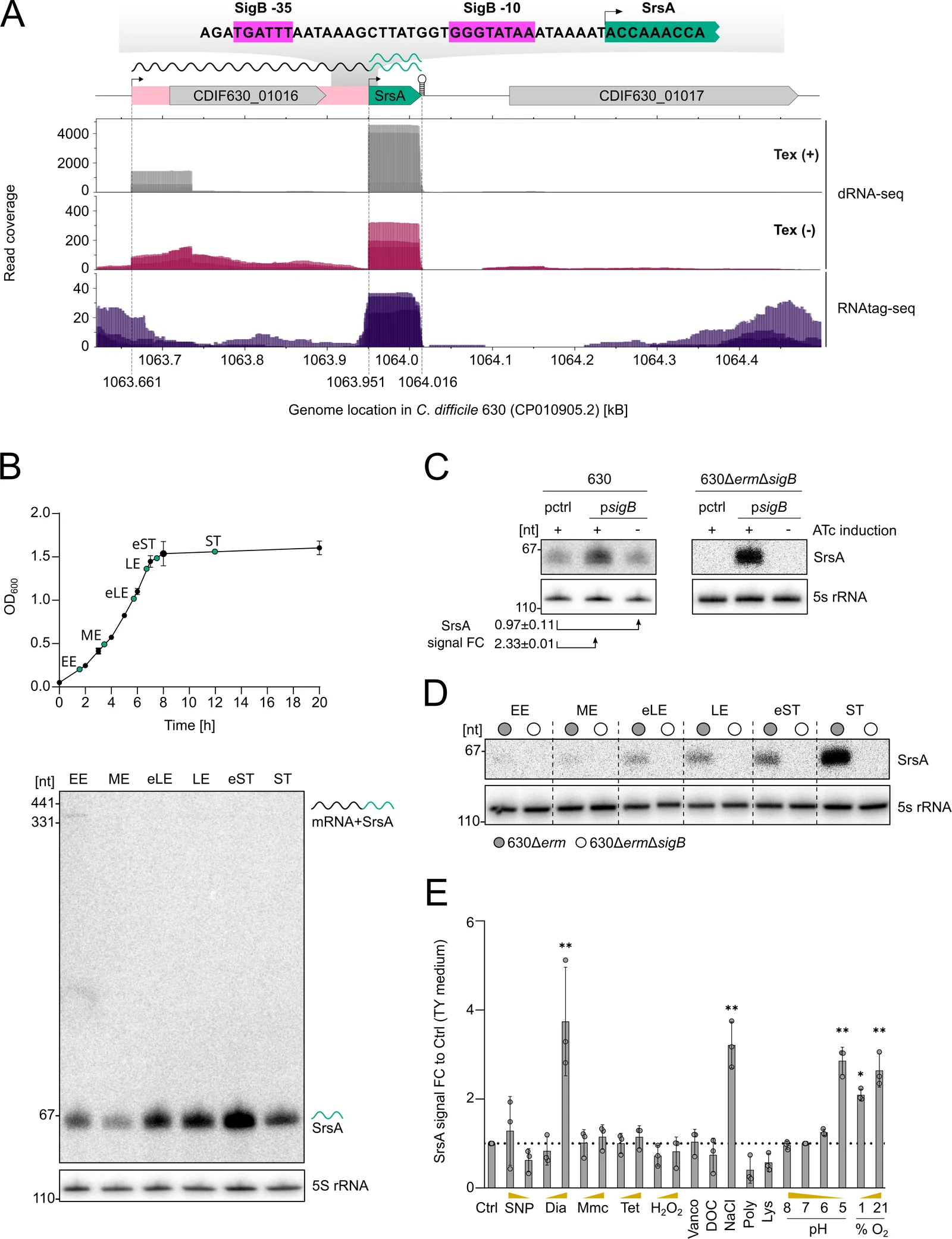
SrsA is transcribed in a σ^B^-dependent manner. (**A**) Read coverage at the SrsA locus and adjacent genes. Tracks show cDNA read counts from differential RNA-seq (dRNA-seq) and RNAtag-seq of *C. difficile* 630 grown to late exponential phase in TY broth (data were generated in reference 18). For dRNA-seq, one fraction of total RNA was treated with terminator exonuclease (Tex+), to degrade processed transcripts carrying a 5′-P or 5′OH. This results in a relative read enrichment of primary transcripts (5′-PPP) in the Tex+-treated libraries (compared to Tex-), allowing the identification of transcription start sites (TSSs). The σ^B^ promoter motif is highlighted in pink, untranslated regions in light red, CDS in gray, and the initial nucleotides of the sRNA sequence in green. TSSs are indicated as arrows and transcription terminators as hairpins. (**B**) Expression profiling of SrsA from *C. difficile* 630 grown in TY medium. The growth curve shows OD_600_ measurements (black circles) and sample collection time points (green circles), representing early exponential (EE), mid-exponential (ME), early late exponential (eLE), late exponential (LE), early stationary (eST), and stationary (ST) phases. Below, the SrsA expression profile detected by northern blot is shown. 5S rRNA serves as a loading control. Data represent the mean ± SD of four biological replicates. (**C**) Northern blot analysis of total RNA from 630 WT and 630Δ*erm*Δ*sigB* cells carrying a multicopy plasmid for anhydrotetracycline-inducible expression of σ^B^ (p*sigB*) or split luciferase (pctrl). Samples were taken after induction of mid-exponential cultures with 100 ng/mL ATc (+) for 1 h. 5S rRNA serves as a loading control. Fold change to the control vector was calculated from three biological replicates, and the mean ± SD is indicated. (**D**) Northern blot analysis of total RNA from 630 WT and 630Δ*erm*Δ*sigB* mutant during growth in TY medium. A representative of three biological replicates is shown. 5S rRNA serves as a loading control. (**E**) Quantification of SrsA expression after 30 min of exposure to a panel of stress conditions, displayed as fold change relative to an unstressed culture (Ctrl). Expression was determined by northern blot analysis of total RNA from cultures in mid-exponential growth phase exposed to indicated stress conditions (SNP, sodium nitroprusside, 50 µM and 10 µM; Dia, diamide, 125 µM and 1,250 µM; Mmc, mitomycin C, 0.625 µg/mL and 2.5 µg/mL; Tet, tetracycline, 4 µM and 16 µM; H_2_O_2_, hydrogen peroxide, 13 µM and 130 µM; Vanco, vancomycin, 2 µg/mL; DOC, desoxycholate; 0.2%; NaCl, 500 mM; Poly, polymyxin B, 70 µg/mL; Lys, lysozyme, 4 mg/mL). Data are shown as mean ± SD from three biological replicates. 5S rRNA serves as a loading control. Significance was determined by ordinary one-way ANOVA with Dunnett’s multiple comparisons test and is indicated as *P < 0.05, **P < 0.01.

In a previous RNA-sequencing-based annotation of sRNAs in the *C. difficile* reference strain 630, we identified SrsA as a putative σ^B^-dependent sRNA (18) because its promoter region contains a highly conserved motif that matches the σ^B^ consensus sequence in *C. difficile* (Fig. 1A). SrsA is encoded in the intergenic region between two hypothetical proteins (Fig. 1A) and is conserved in both genome location and nucleotide sequence, including the promoter region, in strains across all five clades of *C. difficile* (Fig. S1A) (24).

Expression profiling of SrsA in rich medium revealed a prominent ~66-nucleotide-long transcript that is weakly expressed during the early and mid-exponential growth phases but transiently accumulates during the transition to the stationary phase (Fig. 1B). A closer inspection of the transcripts detected by northern blot revealed an additional faint signal of approximately 350 nt during the early exponential growth phase, which matched read-through transcription from a transcription start site (TSS) associated with the gene upstream of SrsA, CDIF630_01016 (Fig. 1A). However, we were unable to reliably detect the upstream mRNA with a probe specific to the CDIF630_01016 coding sequence. Since our dRNA-seq data clearly show that SrsA is transcribed from its own TSS, this suggests that SrsA is primarily transcribed independently of CDIF630_01016 and is therefore a type I 3′ UTR-derived sRNA (25).

To test whether σ^B^ activates SrsA expression, we introduced a plasmid encoding *sigB* under the control of an anhydrotetracycline (ATc)-inducible promoter into *C. difficile* 630 wild-type (WT) and quantified sRNA levels after 1 h of σ^B^ induction during the mid-exponential phase. Northern blot analysis showed a significant increase in SrsA levels compared to the control vector or the uninduced controls (Fig. 1C). Notably, the same experiment in the isogenic 630Δ*erm sigB* deletion background (23) did not reveal any SrsA baseline expression. Yet, ATc treatment still led to strong sRNA induction (Fig. 1C). Finally, to determine whether SrsA transcription would be driven by another sigma factor or a transcription factor, we profiled SrsA levels across different growth phases (Fig. 1D and Fig. S1C). Comparing the 630Δ*erm* and 630Δ*erm sigB* deletion strains indicated that σ^B^ drives SrsA expression under all tested conditions. Notably, slight differences in baseline expression of SrsA in WT backgrounds 630 and 630∆*erm* may arise from growth variations (Fig. 1B and Fig. S1C) or from the previously described point mutation in the peroxide-sensing transcription factor PerR in 630∆*erm* (26). This mutation was shown to cause constitutive derepression of genes involved in the oxidative stress response and to increase oxygen tolerance. Although expression of SrsA in WT 630∆*erm* remains strictly SigB-dependent, we suggest that the loss of PerR repressor activity may contribute to the delayed induction of SrsA expression in WT 630∆*erm* by altering σ^B^ activity.

σ^B^ mediates survival under various stress conditions. To determine whether any of these conditions activate SrsA via σ^B^, we exposed bacteria grown to mid-exponential phase to a panel of stresses, including several conditions known to cause growth defects in the *sigB* mutant (3). This analysis showed a significant induction of SrsA under microaerobic (1%) or aerobic (21%) conditions, low pH, high osmolarity (NaCl), and elevated diamide (Dia) concentrations (Fig. 1E and Fig. S1B). These findings align with the established role of σ^B^ as a key coordinator of the response to oxygen and radical stress in *C. difficile*.

To assess whether SrsA affects bacterial viability under these conditions, we compared the growth of the WT, Δ*srsA*, and Δ*srsA pyrE::PslpA-srsA* (constitutive chromosomal SrsA expression, OE) strains using serial dilution assays on TY agar plates containing various stressors, as described previously (3, 27). This analysis revealed no significant changes in viability under these stress conditions, but Δ*srsA* exhibited slightly reduced fitness under diamide and oxygen stress, as well as at pH 5 (Fig. S1D and E).

In summary, our data indicate that SrsA is part of the σ^B^ regulon in *C. difficile*. SrsA is strongly activated under several exogenous and endogenous stress conditions, including acidic pH, high osmolarity, and oxidative and nitrosative stress. The absence of a clear growth defect under these conditions suggests that SrsA may not directly contribute to the survival responses mounted by SigB but may instead fine-tune other pathways in the cell that facilitate an optimal stress response.

### SrsA exhibits a large Hfq-dependent RNA interactome

**Fig 2:**
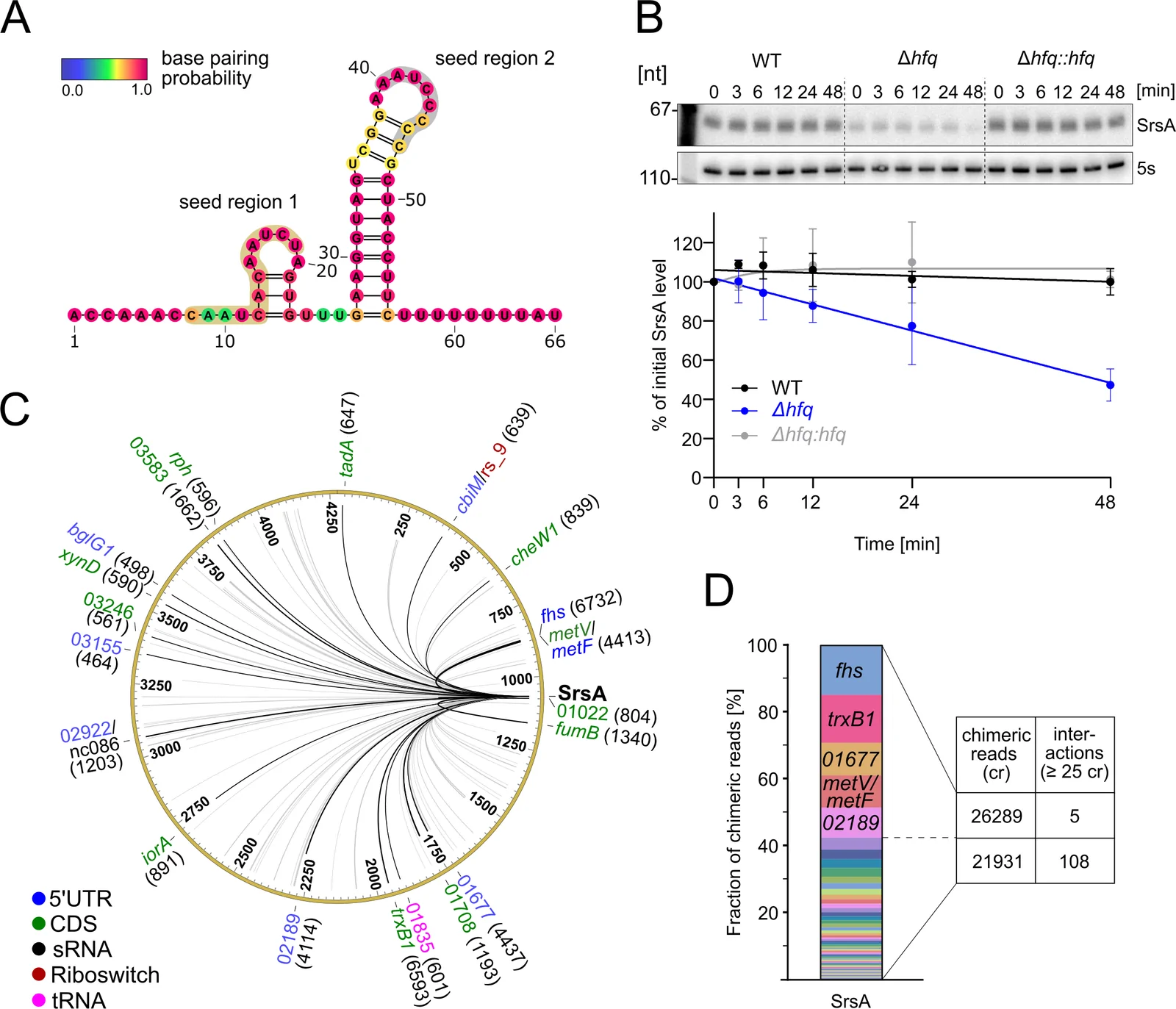
SrsA has a large, Hfq-dependent interactome, with a small overlap to the σ^B^ regulon. (**A**) Secondary structure of full-length SrsA predicted by RNAfold (29). (**B**) Determination of Hfq-dependent SrsA stability. Northern blot analysis of RNA from WT *C. difficile* 630, Δ*hfq*, or the complemented hfq mutant (Δ*hfq:hfq*) grown to the late-exponential phase in TY medium. Total RNA samples were collected at indicated time points after addition of rifampicin to inhibit transcription. One representative northern blot of biological triplicates is shown. SrsA signals were normalized to the 5S rRNA loading control. The mean ± SD of three biological replicates was shown. (**C**) Circos plot representation of the Hfq-dependent SrsA interactome determined by RIL-seq (17). The 20 most abundant SrsA chimeras are labeled with the number of chimeric reads in brackets. Transcript classes/binding regions are color-coded. Edge strength correlates with the number of chimeric reads supporting the interaction. (**D**) Relative distribution of SrsA RIL-seq chimeras. Absolute chimeric read number mapping to the top 5 interactors and the residual interactions involving SrsA are indicated on the right. Interactions below the cutoff (25 chimeric reads) were not considered.

SrsA was previously identified as an Hfq-associated sRNA in *C. difficile* 630 (18). Consistent with this, SrsA displays features of classical Hfq-dependent sRNAs, including an unstructured 5′ region, a stem-loop, and a polyU stretch at the 3′ end (Fig. 2A) (28). Accordingly, an electrophoretic mobility shift assay (EMSA) using *in vitro*-transcribed SrsA and recombinant *C. difficile* Hfq demonstrated formation of a ribonucleoprotein complex (Fig. S2A and B). In gram-negative bacteria, Hfq binding to its sRNA ligands typically contributes to their *in vivo* stability by protecting them from nucleases. Therefore, we tested the impact of Hfq on SrsA stability and steady-state levels by coupling rifampicin-mediated inhibition of *de novo* transcription with northern blot analysis of RNA levels over time. The WT, an isogenic *hfq* mutant (Δ*hfq*), and the complemented *hfq* mutant (Δ*hfq:hfq*) were grown to late-exponential phase, and RNA samples were collected at several time points after rifampicin addition. This revealed a significant reduction in steady-state levels and a decreased half-life in Δ*hfq*, which were restored to WT levels in the complemented strain (Δ*hfq:hfq*) (Fig. 2B).

Having established SrsA as an Hfq-dependent sRNA, we revisited our recent RIL-seq data set for *C. difficile* 630*,* which represents the Hfq-mediated RNA-RNA interactome at the onset of the stationary phase (17). Since this growth phase corresponds to peak SrsA expression, we hypothesized that it would capture a significant fraction of the SrsA interactome. Consistent with this assumption, we identified 113 individual interactions for SrsA (>25 unique chimeric reads) (Fig. 2C and D; Table S1). These interactions primarily involved coding sequences (CDS) and 5′ untranslated regions of mRNAs at various sites, suggesting diverse, target- and site-specific regulatory mechanisms for SrsA (Fig. S2E). In addition, a few interactions involved non-coding transcripts, such as the uncharacterized sRNA CDIF630nc_086 (nc086) and a cobalamin-dependent riboswitch (rs_9) upstream of the *cbiMNQO* operon encoding for a cobalt-specific ABC transporter (Fig. 2C). The five most abundant interactions involved *fhs* and *metF*, which are encoded within the WLP operon (30); *CDIF630_01677* and *CDIF630_02189*, which encode two paralogous small proteins; and the *trxB1* mRNA, which encodes the oxygen-responsive thioredoxin reductase (27). Together, those five interactions accounted for more than 50% of all unique chimeric reads mapping to SrsA (Fig. 2D).

Hfq-dependent sRNAs typically recognize and bind their target transcripts via a short seed region with imperfect complementarity to the target sequence. To narrow down the potential seed region in SrsA, we performed IntaRNA predictions for the top-enriched mRNAs identified by RIL-seq, which revealed two seed regions for SrsA (Fig. 2A). The first seed region (R1) is located in the single-stranded 5′ region of SrsA and overlaps with a small hairpin, whereas the second seed region (R2) is unusual, encompassing a poly-C stretch in the loop region of the intrinsic terminator stem.

Sigma factors are only capable of conferring positive regulation. However, they frequently induce the expression of sRNAs that function as repressors of stress responses by inhibiting the translation of pre-existing mRNAs. The σ^B^ regulon has been thoroughly characterized, most recently through inducible overproduction of σ^B^ combined with differential gene expression analysis, which identified approximately 183 regulated genes (log_2_FC > ±1.5) (23), enabling comparison of both regulons. In fact, the SrsA regulon showed minimal overlap with the σ^B^ regulon, with only seven RIL-seq interaction partners also being differentially expressed upon σ^B^ overproduction (Fig. S2C); six of these carry a σ^B^ promoter motif (Fig. S2D). Therefore, fewer than 10% of the RIL-seq interactions involve σ^B^-dependent gene transcripts.

In summary, SrsA is a typical Hfq-dependent sRNA with minimal overlap with the σ^B^ regulon, suggesting that it primarily coordinates the expression of cellular σ^B^-independent cellular pathways. These pathways may be influenced during stress episodes or may require downregulation to facilitate immediate stress responses.

### SrsA represses a conserved family of membrane-associated small proteins regulating sporulation

To better understand the regulatory function of SrsA, we focused on two of the five most abundant RIL-seq interactions, which involve mRNAs encoding small paralogous proteins belonging to a family of eight conserved small proteins in *C. difficile* 630 (Fig. S3A and B; Table S2) that act as positive regulators of sporulation (31, 32).

**Fig 3:**
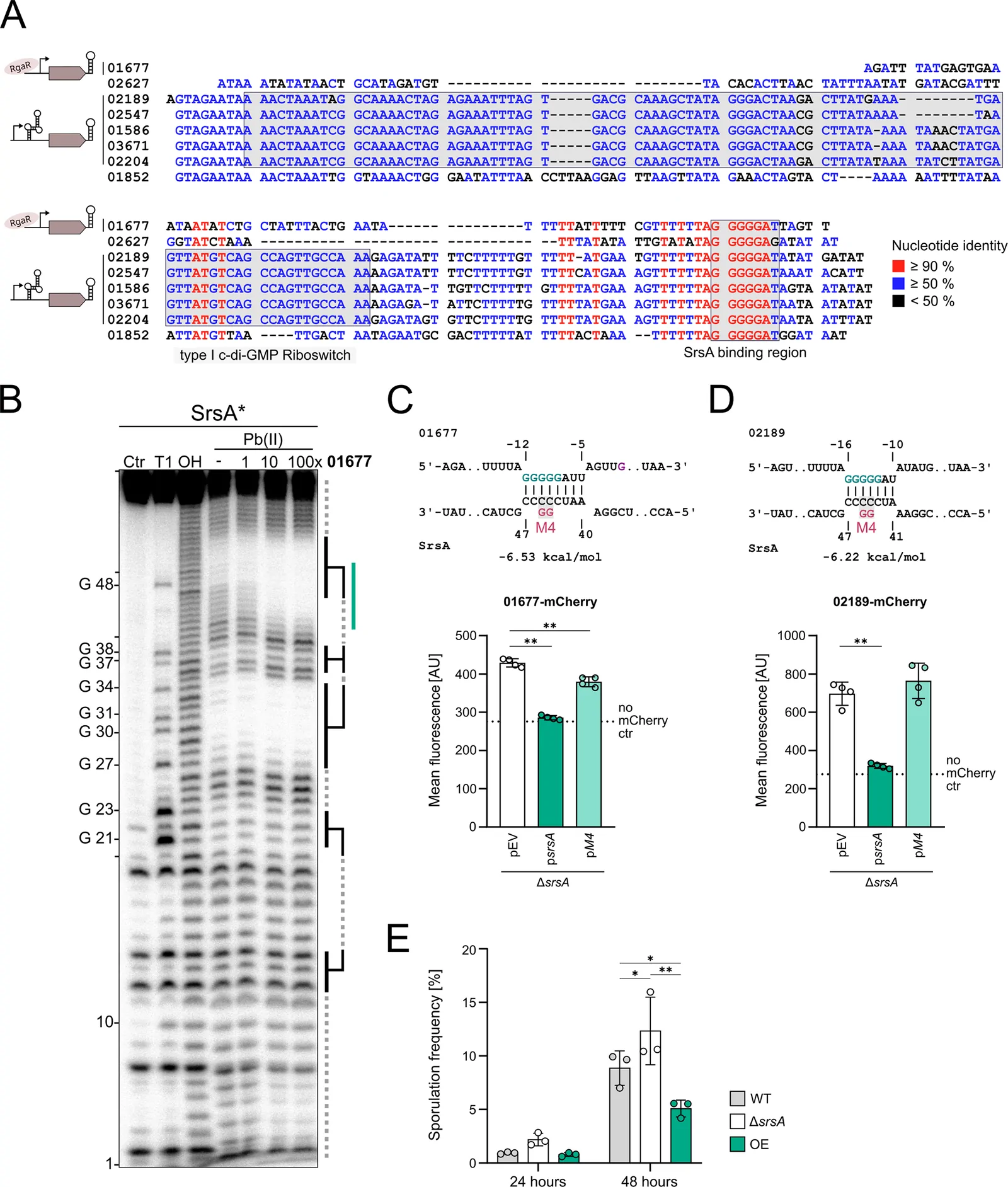
SrsA represses translation of a family of highly conserved small proteins. (**A**) Sequence alignment of the 5′ UTRs from eight genes encoding a family of small paralogous proteins in *C. difficile* 630. The boxed regions indicate a c-di-GMP riboswitch and the predicted sRNA binding site. Alignment was performed with MultAlin (33) using default settings for DNA. The presence of transcriptional (RgaR) and cotranscriptional regulatory elements (c-di-GMP riboswitch) is indicated on the left. (**B**) Lead(II) acetate-based structure probing of ^32^P-5′-end-labeled (*) SrsA in the absence or presence of unlabeled *CDIF630_01677* (*CD1511.1*) mRNA. One representative experiment of three replicates is shown. The predicted secondary structure of SrsA alone is represented by solid black lines for base-paired regions and dashed gray lines for single-stranded regions. The protected site in SrsA is indicated by a green line. The untreated RNA was loaded as a control (Ctrl), the partially RNase T1-digested RNA was used for a G ladder (T1), and a single-nucleotide ladder was created by alkaline hydrolysis of the RNA (OH). Addition of Lead(II) acetate as a cleavage agent is indicated with “Pb(II).” Ratio of unlabeled RNA interactors to labeled RNA in each lane is indicated below. (**C and D**) Predicted base pairing of SrsA at the ribosome-binding sites of *CDIF630_01677* and *CDIF630_02189* is displayed at the top. Nucleotide numbers are indicated relative to the TSS for the sRNA and relative to the start codon for each mRNA. Flow cytometry analysis of translational reporters of *CDIF630_01677* (*CD1511.1*) and *CDIF630_02189 (CD1980.2*) fused to mCherry, measured in arbitrary units (AU), is shown below. Strains carrying only the reporter (pEV) or coexpressing either WT SrsA (p*srsA*) or SrsA with a mutated binding site (p*M4*) were compared with each other. SrsA was constitutively expressed from the *fdxA* promoter, and mCherry fusions were under the control of a tetracycline-inducible promoter. Assays were performed in a Δ*srsA* background. Expression of the target-mCherry reporter was induced with 10 ng/mL anhydrotetracycline for 3 h, starting in mid-exponential phase. The dotted line represents the mean fluorescence intensity of a control strain expressing the sRNA without the mCherry reporter. Data are shown as the mean ± SD of four biological replicates. Significance was determined by one-way ANOVA with Dunnett’s multiple comparisons test and is indicated as **P < 0.01. (**E**) Plate-based assessment of sporulation frequency in 70:30 medium in the 630 wild-type (WT), sRNA deletion (Δ*srsA*), and Δ*srsA pyrE*::P*_slpA_-srsA* (constitutive SrsA overexpression, OE) strains. Data are shown as mean ± SD of three biological replicates. Significance was determined by two-way ANOVA with Tukey’s multiple comparisons test and is indicated as *P < 0.05, **P < 0.01.

The RIL-seq analysis predicted base pairing between SrsA and three of the paralogs—CDIF630_01677 (CD1511.1), CDIF630_02189 (CD1980.2), and CDIF630_01586 (CD1424)—around the conserved Shine-Dalgarno sequence (Fig. 3A). Surprisingly, the most likely interaction between SrsA and these sequences occurs via a poly-C stretch within the intrinsic terminator structure of the sRNA (Fig. 2A and Fig. 3C and D). Lead(II)-based structure probing revealed clear protection of the poly-C stretch in SrsA upon incubation with CDIF630_01677 (Fig. 3B). These results show that SrsA uses unconventional intermolecular base-pairing interactions to regulate its target RNAs.

The high conservation of the target site in CDIF630_01677 suggests that all eight paralogs might be subject to post-transcriptional regulation by SrsA. Interestingly, five paralogs also contain a c-di-GMP I riboswitch in their 5′ UTRs (Fig. 3A), which mediates transcriptional regulation via premature termination (34, 35). To test whether the riboswitch interferes with SrsA regulation, we cloned the 5′ UTRs of CDIF630_01677 (CD1511.1, which lacks the c-di-GMP I riboswitch) and CDIF630_02189 (which harbors the c-di-GMP I riboswitch), along with their initial coding sequences, upstream of an mCherry reporter (Fig. 3C and D). In both cases, fluorescence was significantly reduced when SrsA was co-expressed with the translational reporters, regardless of the presence of the riboswitch. Mutating two cytosines in the predicted seed region of SrsA (“M4”) restored fluorescence levels in both mCherry reporters. This suggests that the presence of the riboswitch has no impact on sRNA-mediated regulation, at least under the tested conditions. Northern blot analysis of CDIF630_02189 mRNA further supported this observation, as SrsA deletion or constitutive overexpression did not affect the premature transcription termination product generated by the CDIF630_02189-associated c-di-GMP riboswitch (Fig. S3C and D). However, this analysis revealed an unexpected decrease of both full-length mRNAs in the absence of SrsA, which was reversed by constitutive SrsA overexpression, suggesting additional effects of the sRNA, for example, on mRNA stability (Fig. S3C and D). Therefore, we assessed the effect of SrsA on relative protein expression of chromosomally 3×FLAG-tagged CDIF630_02189. This indicated that constitutive expression of SrsA results in approximately a 2-fold reduction in protein levels compared with an empty vector control (Fig. S3E and F).

Overall, these data identify a repressor function of SrsA for two of the small protein paralogs and further demonstrate that the CDIF630_02189 mRNA is regulated by SrsA and the riboswitch through distinct mechanisms. This is reminiscent of the *btuB* mRNA in *E. coli,* which is selectively regulated by an adenosylcobalamin-responsive riboswitch and by the OmrA and OmrB sRNAs (36). Given the conserved SrsA target site in all eight paralogs, our data further suggest that SrsA may regulate all eight paralogs, potentially under distinct, yet unknown, conditions.

A recent study demonstrated that CDIF630_02189 overexpression induces early sporulation genes, while the deletion of seven of the small proteins, including CDIF630_02189 and 01677, strongly decreases the sporulation frequency compared to the WT, overall assigning a sporulation-promoting role to this family of small proteins (31, 32). Accordingly, a sporulation assay in liquid 70:30 medium using the wild type (WT), Δ*srsA*, and the chromosomal SrsA overexpression (OE) showed that deletion of *srsA* results in slightly increased sporulation frequency (significantly at the 48-h time point), while sRNA overexpression reduced sporulation frequency (significantly at the 48-h time point) compared with the WT (Fig. 3E). In summary, our results demonstrate that SrsA suppresses spore formation likely through translational inhibition of several, if not all, paralogous mRNAs during σ^B^-inducing stress conditions, which aligns with previous studies showing that a *sigB* deletion results in increased sporulation frequencies (3, 37).

### SrsA base pairs with multiple target sites in the polycistronic WLP operon

**Fig 4:**
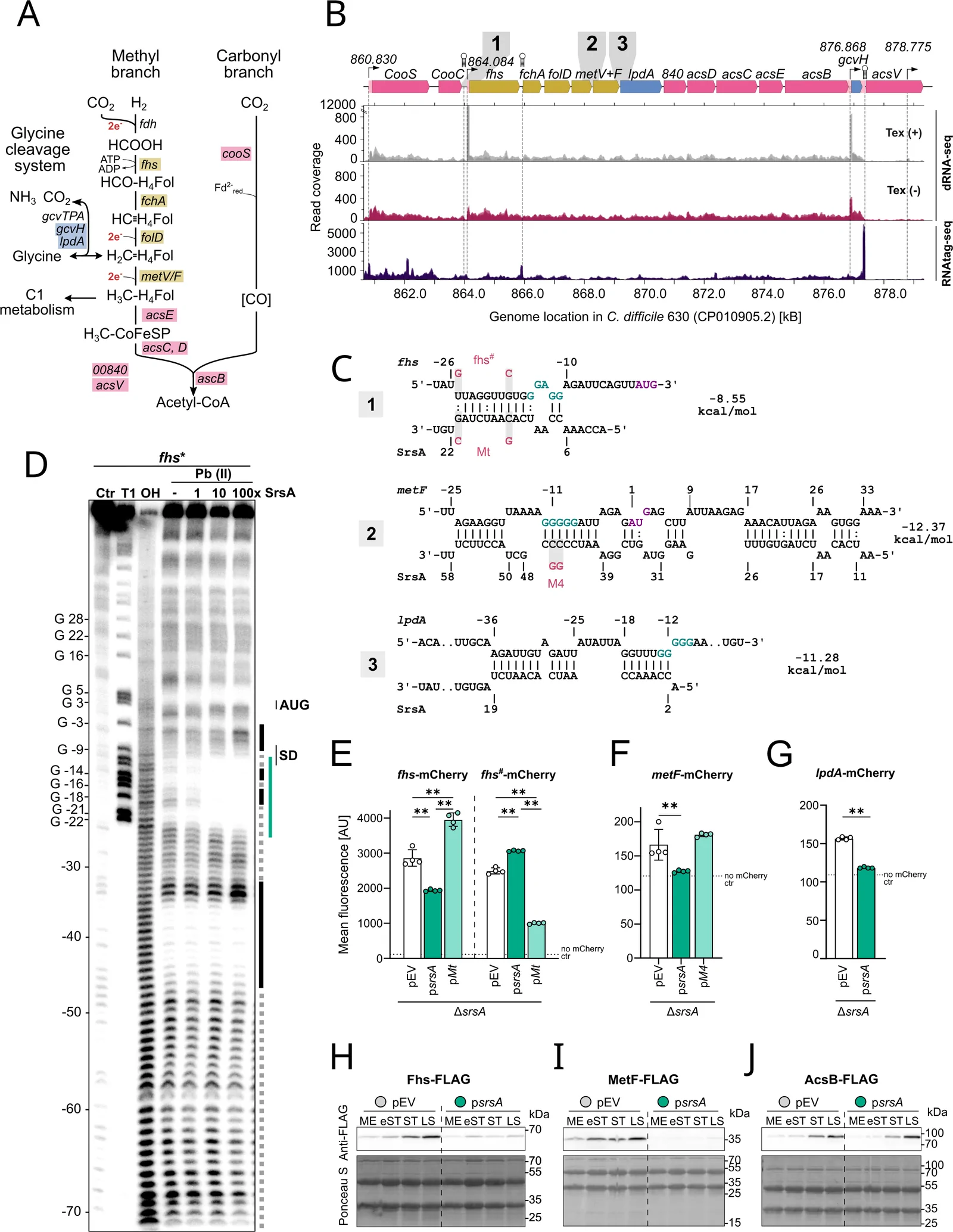
SrsA interacts with the ribosome-binding sites of *fhs*, *metF,* and *lpdA* in the Wood-Ljungdahl operon to repress their translation. (**A**) Schematic representation of the metabolic Wood-Ljungdahl Pathway (WLP), including the methyl and carbonyl branches (genes highlighted in gold and violet, respectively), and interconnected pathways such as C1 metabolism and the glycine cleavage system (genes highlighted in blue). HCOOH, formate; HCO-H_4_Fol, *N*^10^-formyl-THF; CH≡ H_4_Fol, *N*^5^,*N*^*10*^-methenyl-THF; CH_2_=H_4_Fol, methylene-THF; CH_3_-H_4_Fol, methyl-THF; H_3_C-CFeSP, methyl bound to corrinoid catalytic center of AcsC; [CO], carbonyl-intermediate generated by CooS. (**B**) Genomic organization and read coverage represented by cDNA read counts for differential RNA-seq (dRNA-seq) and RNAtag-seq at the WLP operon locus. The primary transcriptome architecture of the WLP was determined by differential RNA-seq (dRNA-seq) and RNAtag-seq during the late exponential phase in TY medium (data were generated in reference 18). Bent black arrows indicate TSS; hairpins indicate Rho-independent transcription termination sites (based on RNAtag-seq peaks and mRNA secondary structure predictions). (**C**) Predicted base pairing of SrsA with the *fhs*, *metF,* and *lpdA* mRNAs. Nucleotide numbers are indicated relative to the TSS for the sRNA and relative to the start codon for the mRNA. The Shine-Dalgarno sequence is indicated in teal and the start codon in violet. The approximate binding site within the whole WLP operon is highlighted in panel **B **with bold numbers. Seed region mutations in the sRNA (Mt and M4) and compensatory mRNA mutations (fhs^#^) used in the reporter system in panel **E **are indicated in red. (**D**) Lead (II)-based structure probing of ^32^P-5′-end-labeled (*) 5′ UTR of *fhs* (from TSS to + 78 nt) in the absence or presence of unlabeled SrsA. One representative of three replicates is shown. *In silico* predicted secondary structure of 5′ UTR of *fhs* alone is represented as black continuous lines (base paired regions) and dashed gray lines (single stranded regions). Interaction sites for SrsA are shown as a green line. (**E–G**) Flow cytometry analysis of translational reporters of *fhs*, *metF,* and *lpdA* fused to mCherry measured in arbitrary units (AU). SrsA WT (p*srsA*) and mutant (pMt or pM4) strains were compared to the reporters without sRNA overexpression (pEV). SrsA was constitutively expressed from the *fdxA* promoter, and mCherry fusions were under the control of the moderate, constitutive *cwp2* promoter. Assays were performed in a Δ*srsA* background. The dotted line represents the mean fluorescence intensity of the strain expressing the sRNA without the mCherry reporter. The mutated *fhs* reporter is marked with a hashtag, and mutation sites are indicated in red font with gray background in panel **C**. The mean ± SD of four biological replicates is shown. Significance was determined by ordinary one-way ANOVA with Tukey’s multiple comparisons test and is indicated as **P < 0.01. (**H–J**) Protein levels of chromosomally 3×FLAG-tagged Fhs (**H**), 3×FLAG-tagged MetF (**I**), and 3×FLAG-tagged AcsB (**J**) in strains carrying a plasmid constitutively expressing SrsA (p*srsA*) or an empty vector control (pEV). Samples were taken during mid-exponential (ME, 4 h), early stationary (eST, 8 h), stationary (ST, 12 h), and late stationary phase (LS, 24 h). Whole-protein staining with Ponceau S serves as a loading control. One representative blot of four biological replicates is shown.

We then examined the second set of highly abundant SrsA interactions identified by RIL-seq, which involves genes in the Wood-Ljungdahl pathway (WLP). The WLP has been proposed to be important for gut colonization by supporting efficient NAD^+^ regeneration in the absence of Stickland acceptor amino acids such as proline, leucine, and glycine (30, 38). It is an ancient metabolic pathway that fixes CO_2_ and progressively reduces it to acetyl-CoA, which can be converted to acetate or used for biosynthetic purposes (Fig. 4A).

The WLP is encoded by a 15-gene locus that contains all genes required for the entire pathway (30), but despite its predicted central metabolic function in *C. difficile*, little is known about its molecular regulation or the environmental triggers that activate or repress it. Interestingly, our previous transcriptome annotation of *C. difficile* 630 identified three transcription start sites (TSSs) within the predicted multi-cistronic operon and three transcription termination sites (Fig. 4B) (18). Together with the sRNA regulation identified here, this suggests that the WLP may be expressed as several distinct sub-operons to fine-tune the production of enzymes from the methyl-fixing and carbonyl-fixing branches, with additional post-transcriptional fine-tuning in response to distinct stress conditions.

*In silico* target predictions using IntaRNA identified potential binding sites in the 5′ UTRs of *fhs* and *metF*, as well as an additional predicted interaction with *lpdA*, each overlapping the Shine-Dalgarno sequence of the respective genes (Fig. 4C). To confirm the predicted base pairing, we first performed EMSAs combining radioactively labeled SrsA with *in vitro*-transcribed mRNA fragments containing the putative target sites. This indicated that SrsA forms high-affinity complexes with the translation initiation regions of *metF* and *lpdA* and a moderate-affinity complex with *fhs* (Fig. S4A through C). Lead (II)-based structure probing of the radioactively labeled 5′ UTR region and initial CDS of *fhs* indicated efficient protection of the translation initiation region in the presence of the sRNA (Fig. 4D, green line). A reciprocal experiment with radioactively labeled SrsA demonstrated that *fhs* mRNA interacts with the largely unstructured seed region of SrsA, ranging from nucleotide 6 to 21 (Fig. S4D, red line). Similarly, the predicted base pairing of the 5′ terminal unstructured region of SrsA to *lpdA* was confirmed by lead (II)-based structure probing (Fig. S4F), further supporting the primary interaction motif suggested by RIL-seq.

The *in silico* predicted interaction of SrsA with *metF* not only includes the single-stranded seed region of the sRNA but extends into the stem loop (Fig. S4E), implying that base pairing of SrsA with *metF* will result in unfolding of the sRNA structure. Indeed, lead (II)-based structure probing revealed a global rearrangement of SrsA structure upon incubation with *metF* (Fig. S4E, red line).

In line with a potential repressive effect of SrsA on these WLP enzymes, mRNA levels of *fhs* and *metF* were reduced upon SrsA overexpression and slightly increased in the ∆*srsA* mutant, especially during early stationary phase (Fig. S5A). To test whether the sRNA interactions with *fhs*, *metF*, and *lpdA* lead to post-transcriptional regulation of mRNA translation, we fused their 5′ UTRs and initial coding sequences to mCherry. Co-expressing SrsA with the *fhs* reporter from a plasmid reduced translation compared to that of the reporter alone (Fig. 4E). Mutating either the SrsA seed region (Mt) or the *fhs* binding site (*fhs*^#^) (Fig. 4C) disrupted regulation, whereas restoring the interaction by mutating both reconstituted the regulatory effect, confirming that base pairing of SrsA with *fhs* downregulates translation (Fig. 4E). Likewise, the fluorescence of the *metF* (Fig. 4F) and *lpdA* (Fig. 4G) reporters was diminished in the presence of SrsA, and mutation of the corresponding seed region in SrsA abolished *metF* repression.

Post-transcriptional regulation of individual genes within polycistronic operons can yield distinct regulatory outcomes (39). In certain circumstances, it may allow fine-tuning of the stoichiometry of individual enzymes within a biosynthetic pathway (discoordinate regulation) (40). More commonly, it leads to general repression and destabilization of the entire mRNA (coordinate regulation). Determination of target RNA stability within the operon indicated that constitutive SrsA expression destabilized them compared to the WT, whereas SrsA deletion had moderate effects (Fig. S5A). Monitoring protein production in WT and sRNA overexpression strains using chromosomally 3×FLAG-tagged *fhs*, *metF*, and *acsB* (Fig. 4H through J) revealed a continuous increase in all three proteins from exponential to stationary phase in the WT. By contrast, plasmid-based constitutive expression of SrsA strongly decreased protein levels of its direct targets, Fhs-3×FLAG and MetF-3×FLAG, across all tested growth phases, whereas AcsB-3×FLAG was only mildly downregulated (Fig. 4H through J and Fig. S5B).

Taken together, we show that SrsA base pairs with multiple sites in the polycistronic WLP mRNA, thereby mediating an overall downregulation of enzyme levels in the WLP.

### SrsA contributes to disease severity in a mouse model of *C. difficile* infection

**Fig 5:**
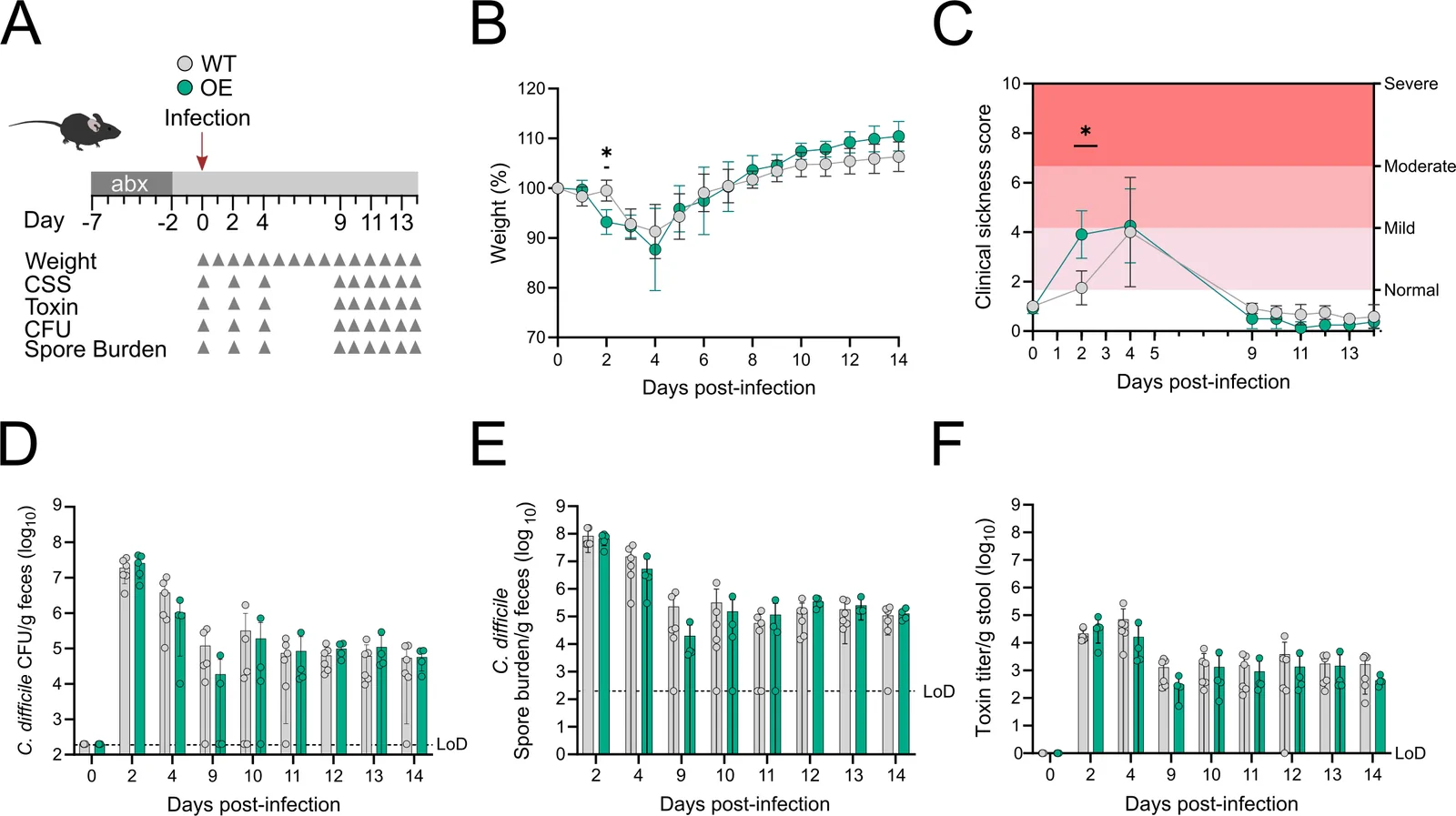
SrsA enhances pathogenicity in a mouse model of *C. difficile* infection. (**A**) Schematic of mouse model of infection. Cefoperazone was supplemented in the drinking water *ad libitum* for 5 days (abx) to make mice susceptible to infection. After a 2-day recovery period, mice were challenged with 10^4^ spores of *C. difficile* 630 wild-type (WT) or Δ*srsA pyrE::*P*slpA*-srsA (OE). Mouse weight and clinical sickness score (CSS), as well as *C. difficile* bacterial burden (CFU), toxin titer, and spore burden from stool, were assessed at select time points (gray triangles). (**B and C**) Mouse weight (**B**) and clinical sickness score (CSS) (**C**) were monitored throughout the course of infection. (**D–F**) *C. difficile* bacterial burden (CFU) (**D**), spore burden (**E**), and toxin titer (**F**) were quantified in stool at the indicated time points. Data are shown as mean ± SD, and statistical significance was determined by two-way ANOVA with multiple Mann-Whitney tests in all graphs and is indicated as *P < 0.05. Dotted lines in panels **D–F **indicate the limit of detection.

A *sigB* mutant is significantly attenuated in a mouse infection model (3). To determine whether SrsA contributes to colonization and disease, mice were orally challenged with 10^4^ spores of the WT or a strain constitutively expressing SrsA from the *pyrE* locus (OE), following cefoperazone administration in the drinking water to induce susceptibility to infection (Fig. 5A) (41, 42). Over 14 days, we monitored clinical sickness, a composite score of mouse weight, stool quality, and behavior, as well as *C. difficile* burden, spore burden, and toxin titer in the feces. Infection with both strains resulted in weight loss and an increased clinical sickness score, but OE-infected mice experienced greater weight loss and clinical sickness than WT-infected mice on day two post-infection (Fig. 5B and C). While all WT-infected mice survived the entire course of infection, two mice infected with the OE strain either died or had to be euthanized due to weight loss (Fig. S6G). Despite these differences in disease severity, *C. difficile* bacterial burden, fecal spore shedding, and toxin titer were similar among both infected groups (Fig. 5D through F).

A hallmark of *C. difficile* infection (CDI) in the clinic is its high recurrence rate, observed in approximately 20% of patients (43). Moreover, relapsing infection after antibiotic treatment represents a bottleneck for *C. difficile* in the gut. Thus, to further assess the contribution of SrsA to disease recurrence, we used a relapse model of CDI utilizing the same WT and OE strains (44). Both infection groups were treated with a 5-day course of the antibiotic vancomycin (vanco) in the drinking water to induce relapse (Fig. S6A). Following antibiotic treatment, mice were placed on regular water and monitored for signs of relapse infection, such as weight loss (Fig. S6B), increased clinical sickness (Fig. S6C), and the reappearance of *C. difficile* colony-forming units (CFUs) and toxin in the stool. Here, a pattern similar to the standard infection model was observed, as mice infected with OE experienced three deaths, while all mice from the WT-infected group survived (Fig. S6G). Both infection groups cleared toxin after vancomycin treatment, and all WT-infected mice also cleared *C. difficile* CFUs on days 11 and 12 post-infection. Conversely, three OE-infected mice did not fully clear *C. difficile* during this period. (Fig. S6D through F).

Together, these data suggest that SrsA contributes to disease severity and gut colonization, particularly after antibiotic-induced population bottlenecks that impose various stresses on the bacteria. Although the precise mechanism remains unknown, it likely involves reinforcing the adaptive physiological and stress responses mounted by σ^B^ during this critical infection phase.

## DISCUSSION

In this work, we expand the regulon of the alternative σ factor σ^B^ by identifying a small RNA that serves as the non-coding arm of this stress response. σ^B^ is a widely conserved sigma factor in gram-positive bacteria that coordinates responses to various stress conditions. However, the specific genes it regulates and the inducing conditions vary by bacterial species and are tailored to each species’ natural habitat. In the case of *C. difficile*, a key function of the regulon appears to be the response to oxidative stress (23). Consistent with this established role of σ^B^, we show that SrsA is strongly induced in response to oxidative stress and molecular oxygen, as well as osmotic stress, acidic pH, and during the transition to the stationary phase of growth. The RIL-seq analysis that identified SrsA targets was performed at transition to stationary phase, a time window in which SrsA expression naturally peaks during *in vitro* growth. Consistent with this peak expression, we capture a large SrsA interactome comprising 113 interactions between SrsA and individual transcripts. Given the associated growth phase, these components likely reflect regulatory adaptations to nutrient limitation and to mounting stationary-phase processes, such as sporulation. Indeed, the most abundant interactions involved genes in the WLP operon, which is transcriptionally upregulated in stationary phase (Fig. 4H through J), and may reflect a metabolic response to the depletion of the reductive Stickland acceptors proline, glycine, and leucine, which facilitate recovery of NAD^+^ and synthesis of ATP during exponential growth (30, 38, 45). The other top-enriched transcripts that were also validated in this study comprised the conserved, paralogous proteins CDIF630_01677 and CDIF630_02189. Both mRNA and protein levels of the tested paralogs increase during the transition from exponential growth to the onset of the stationary phase, which, together with their role in regulating sporulation, links them to stationary phase processes (32).

### SrsA links the stress response to the regulation of sporulation

Earlier studies showed that a *sigB* deletion leads to increased sporulation efficiency (3, 46) and suggested that this increase in sporulation is due to the modulation of Spo0A phosphorylation by σ^B^-regulated kinases. Our data, showing that deletion of *srsA* leads to increased sporulation frequency, suggest that σ^B^-induced SrsA provides another mechanism that contributes to the increased sporulation frequency. We propose that this sporulation phenotype may be the result of SrsA-mediated post-transcriptional regulation of a family of eight small proteins that promote sporulation (31, 32). Specifically, the stepwise deletion of seven members leads to a proportional decrease in sporulation frequency, and ectopic overexpression of CD1980.2 (CDIF630_02189) promotes hypersporulation and increased expression of early sporulation genes (32).

Our data demonstrate direct binding of SrsA to the translation initiation regions of CDIF630_01677 (CD1511.1) and CDIF630_02189 (CD1980.2), resulting in their translational inhibition. Based on the high sequence conservation of the ribosome-binding site region of these genes, SrsA may be able to repress translation of all paralogs. This negative regulation coincides with a reduced sporulation frequency in an SrsA overexpression strain. Combined, these independent findings suggest that reduced levels of the small proteins, either by genetic deletion or by post-transcriptional repression via SrsA, result in reduced sporulation efficiency.

Intriguingly, expression of all protein paralogs appears to be regulated by environmental signals. The RgaR/S two-component system regulates CDIF630_01677 (CD1511.1), and five of the remaining paralogs contain a type I c-di-GMP riboswitch in their 5′ UTR (Fig. 3A). Finally, regulation via SrsA links them to specific stress conditions relevant during gut colonization. This suggests that these proteins may function as regulatory hubs that integrate physiological cues into the regulation of sporulation.

In addition to the conserved small proteins, interactions of SrsA with other sporulation genes were predicted by the RIL-seq data set. This comprises the SigE-dependent *spoIVA* mRNA (79 chimeric reads), which encodes an ATPase that serves as a scaffold for the spore coat proteins and is required for their proper localization within the mother cell (47–49). Furthermore, SrsA is predicted to bind the *sigG* mRNA (112 chimeric reads), which encodes the sigma factor that coordinates late-stage sporulation events in the forespore (50, 51). In addition, we identified chimeras with the mRNA encoding a putative *tepA* homolog (25 chimeric reads) and the CDIF630_02895 mRNA (112 chimeric reads), a putative sporulation protein encoded in the operon adjacent to *sigG*. Therefore, SrsA may modulate the expression of sporulation genes important for spore coat assembly and sporulation coordination downstream of *spo0A*. However, since all four interactions occur more than 150 nucleotides downstream of the start codon, canonical regulation of translation initiation appears unlikely, warranting further experimental validation.

### Functional implications of post-transcriptional regulation of the WLP

In *C. difficile,* the main function of the WLP, which is also known as the reductive acetyl-CoA pathway, is likely to serve as an electron sink during heterotrophic growth. The regeneration of NAD^+^ from NADH via the WLP allows the utilization of simple sugars such as glucose and may optimize ATP yield during fermentation (30, 38). The WLP represents not just a metabolic strategy to fix CO_2_ for the synthesis of acetyl-CoA but also connects to other vital cellular functions. The first enzymatic steps in the WLP operon contain components of the glycine cleavage system (*lpdA* and *gcvH*, Fig. 4A), suggesting that *C. difficile* can use CO_2_ fixation for the synthesis of glycine. In the autotrophic bacterium *C. drakei*, which has a similar operon structure, it has been demonstrated that the methyl-fixing branch of the WLP can be harnessed for glycine synthesis via the glycine cleavage system, which increases metabolic flexibility (52). Additionally, the methyl-fixing branch contributes to the synthesis of methyl-THF (via MetV/MetF), which serves as an important C_1_ donor for various essential processes, including methionine synthesis (53). In light of this, it is not surprising that gene essentiality screens in *C. difficile* 630 and R20291 identified two genes of the methyl-fixing branch as essential (*fhs* and *folD*) and a third (*fchA*) as an ambiguous candidate (54, 55). Hence, the sRNA-based regulation of the WLP mRNA may provide an efficient strategy to allow the time-constrained, conditional repression of the essential genes in the WLP.

Under which conditions might this post-transcriptional downregulation be necessary? This question is relevant because the peak expression of SrsA at the onset of the stationary phase (Fig. 1B) coincides with upregulation of the WLP (Fig. 4H through J). It seems counterintuitive that the expression of the repressor coincides with the expression of the target transcript. However, SrsA expression is transient during the onset of the stationary phase (Fig. 1B) and may help to fine-tune the levels of WLP enzymes during a transition from reductive Stickland reactions to alternative NAD^+^ regenerating pathways. Another scenario may involve the response to oxidative stress, which was a strong and rapid inducer of SrsA expression (Fig. 1E). Approximately a third of the σ^B^ regulon consists of oxidoreductases that have the potential or have been shown to participate in the detoxification of reactive oxygen species, including O_2_^−^, H_2_O_2_, and OH^−^ through various mechanisms (56–58). Importantly, all these mechanisms include the use of reduced electron carriers, such as NADH, NADPH, or ferredoxins, from which electrons are either directly transferred to the reactive species or to an oxidized protein residue. In the related anaerobe *Clostridium acetobutylicum*, H_2_O_2_-induced oxidative stress results in a reduced NADH/NAD^+^ ratio, consistent with the idea that reduction equivalents are employed for oxygen detoxification. This, in turn, activates the Rex transcriptional regulator, which helps to preserve or recover reduction equivalents by repressing reductive pathways (59). By targeting *fhs* and *metF*, SrsA represses the ATP-consuming conversion of formate to formyl-THF, as well as the NADH-consuming reduction of methylene-THF to methyl-THF (Fig. 4A). This implies that SrsA-driven repression of the WLP could bolster oxidative stress defenses by maintaining reduction equivalents such as NADH. Therefore, sRNA-mediated repression of reductive metabolic pathways may represent one function of the noncoding arm of the σ^B^ response that supports the oxidative stress defense by oxidoreductase enzymes.

### Challenges of correlating *in vivo* and *in vitro* fitness

Our study identified both distinct stress conditions that activate SrsA expression via the stress response sigma factor B and regulated pathways; yet, aside from sporulation, we could not clearly identify pronounced fitness defects associated with *srsA* deletion. This is partly due to the challenge of simulating *in vitro* conditions that force *C. difficile* to grow exclusively using the Wood-Ljungdahl pathway and subsequently demonstrate a growth phenotype. Since several WLP genes (*fhs*, *folD*, and likely *fchA*) are essential, we were unsuccessful in generating a WLP knockout. As a workaround, an *acsB* mutant lacking a subunit of acetyl-CoA synthase was successfully generated in a previous study (30). AcsB (as a proxy for WLP activity), however, is produced only in the absence of amino acid Stickland acceptors proline, glycine, and leucine (30), a condition that hardly supports growth of *C. difficile in vitro*, making it difficult to assess any growth defects of the *srsA* mutant. On the other hand, transcriptomic analyses in infected mice show that WLP genes are highly expressed during gut colonization, an environment unlikely to be depleted of all amino acid acceptors (60). This indicates that *C. difficile* faces *in vivo* conditions that are hard to replicate *in vitro*, as highlighted by our inability to clearly identify a single stress condition under which SrsA activity is required for survival or continued growth.

Therefore, we assessed the role of SrsA in *C. difficile* colonization and disease using a mouse model of both primary and relapse infection, given that high recurrence rates are a major clinical challenge. Though the *in vivo* colonization phenotypes are also subtle, we observed differences in disease severity and bacterial recovery between WT-infected mice and mice infected with the SrsA overexpression strain. These differences were most pronounced in the relapse infection model, in which the bacteria experience a strong population bottleneck after antibiotic treatment. Here, the mice infected with the SrsA OE strain did not fully clear the bacteria after antibiotic treatment and showed a lower survival rate during relapse infection, suggesting that SrsA contributes to bacterial colonization and disease. Since we did not find any differences in toxin levels between the infection groups, more studies are needed to understand the underlying mechanisms of this increased disease severity.

### The RIL-seq data set suggests an unconventional mode of action for SrsA regulation

Stress-associated sRNAs are best characterized for the σ^E^-mediated envelope stress responses in the Proteobacteria *S. enterica*, *E. coli,* and *V. cholerae*. Here, the coding arm, mediated by σ^E^, promotes the refolding or degradation of misfolded proteins, while the non-coding arm, mediated by several associated sRNAs, provides the stress response with a repressor function that inhibits the translation of novel OMPs to prevent additional accumulation of misfolded proteins (4, 5, 8). In gram-positive bacteria, RsaA from *S. aureus* is the only σ^B^-dependent sRNA with experimentally validated targets (11, 12). Here, too, the sRNA acts as a translational repressor of several mRNAs, including that of MgrA, a global transcriptional regulator of biofilm formation and capsule production. Our functional characterization of SrsA likewise reveals that it serves as a canonical repressor of mRNA translation through binding to the RBS region of the here validated transcripts. However, a closer inspection of the SrsA interaction sites in the RIL-seq data set shows that most are found within the coding sequences of mRNAs (Fig. S2E). This indicates that currently less appreciated regulatory mechanisms may play a significant role in SrsA-mediated regulation of stress responses in *C. difficile*. Recently, it was shown that binding of the *E. coli* sRNA ArcZ within coding sequences provides an inducible mechanism to enhance the enzymatic activity of the produced proteins, likely by improving cotranslational protein folding (61). Functional characterization of the σ^E^-dependent FoxJ sRNA in *F. nucleatum* also revealed additional, unique regulatory properties, including transcriptional activation of target transcripts (7). Therefore, SrsA-assisted modulation of translation speed could provide an effective means for proteins to adapt to stress, particularly oxidative stress, which can impact protein synthesis at multiple levels (62). Interestingly, among the SrsA-dependent targets, we find σ^B^-transcribed genes, and here, too, the SrsA interaction occurs at sites deep in the coding sequence (*trxB1*, *trxB2*, *uvrC*, *xynD*, *CDIF630_01627*), upstream of the ribosome-binding site (*recA*, *CDIF630_01709*), or in the 3′ UTR (*xynA*). Altogether, this suggests that SrsA activity may synergize with σ^B^ to optimize production of σ^B^-dependent proteins through unconventional regulatory mechanisms.

### Outlook

Here, we have characterized the regulon of SrsA at the onset of the stationary phase, a condition most likely reflecting nutrient limitation as the major stressor. However, we also identified several SrsA-inducing stress conditions that are relevant during gut colonization—most notably oxygen. Fluctuations in oxygen levels frequently occur during infection and may therefore be a major activator of SrsA expression *in vivo*. It is likely that parts of the SrsA regulon will be rewired in response to distinct stress conditions to mediate optimal adaptation to the specific challenges associated with different stresses, or a combination of them. Therefore, mapping the targetome of SrsA under these stress conditions will likely provide novel insights into aspects of the σ**^B^** stress response that were not previously considered.

Furthermore, SigB was shown to be expressed in a bimodal fashion, being active in only a subset of cells within a population. This suggests that SrsA expression and regulation might also occur in only a subset of cells. Single-cell approaches such as RNA FISH or single-cell RNA-seq might provide valuable insights needed to better understand potential heterogeneity of this non-coding regulator and how this heterogeneity contributes to stress responses on a population level.

In summary, the regulatory outputs of stress-associated sRNAs might be more diverse than initially anticipated, extending well beyond the canonical mechanism of translational repression, and likely contribute to phenotypic heterogeneity. Future efforts should focus on these yet unexplored mechanisms to increase our mechanistic understanding of bacterial adaptive stress responses.

## MATERIALS AND METHODS

### Bacterial culture conditions

*C. difficile* strain 630 (DSM 27543) was cultured at 37°C inside a Coy chamber, using an anaerobic gas mix (80% N_2_, 10% H_2_, 10% CO_2_). Strains were grown on brain heart infusion (BHI) agar plates (1.5% agar) or in autoclaved BHI broth (37 g/L), unless indicated otherwise. Agar plates were incubated anaerobically for at least 4 h prior to inoculation, while liquid media were kept in the Coy chamber overnight to allow diffusion of entrapped oxygen. Plasmid-harboring *C. difficile* 630 were streaked on BHI agar containing 15 µg/mL thiamphenicol, 8 µg/mL cefoxitin, and 250 µg/mL cycloserine and grown in the presence of 15 µg/mL thiamphenicol in broth cultures. Cryostocks were stored at −80°C in BHI broth containing 15% glycerol.

### Plasmid construction

A list of all oligonucleotides used in this study can be found in Table S3.

### Plasmids for chromosomal manipulation

#### pFF-103: construct for chromosomal *srsA* deletion

For deletion of *srsA*, homology arms flanking the region from −35 nucleotides to +10 nucleotides relative to the TSS were amplified from *C. difficile* 630 gDNA (FFO-640/641 and FFO-642/-643) and joined by SOEing PCR (FFO-640/643). The pMTL-SC7315 (63) vector backbone was PCR-linearized (FFO-362/363), and the vector and homology arms were ligated using the Gibson Assembly Master Mix from NEB according to the manufacturer’s instructions, resulting in pFF-103.

#### pFF-488: construct for SrsA overexpression from neutral *pyrE* locus

A constitutive overexpression of SrsA driven by the *slpA* promoter and carrying an additional slpA terminator at the 3′ end was generated for insertion at the neutral pyrE locus.

To generate this plasmid, SrsA with an ensuing slpA terminator was amplified from pFF-36 (FFO-1844/1410). pFF-36 was generated by PCR-linearizing pRPF185 (64) (FFO-323/324) and inserting SrsA (amplified from gDNA with FFO-335/336) between the *tetR-P_tet_* system and the slpA terminator by Gibson assembly. To add the *slpA* promoter, the 100-nucleotide region upstream of the *slpA* transcription start site was amplified from *C. difficile* 630 gDNA (FFO-1016/1843) and appended to the *srsA-slpA* terminator fragment by SOEing PCR (FFO-1016/1410). The product was inserted into pJAK080 (18) using the KpnI and BamHI restriction sites, generating pFF-488.

#### pFF-430, pFF-501, pFF-502, and pFF-596: constructs for chromosomal 3×FLAG tagging of *acsB*, *fhs*, *metF*, and CDIF630_02189

To generate C-terminally 3×FLAG-tagged versions of *acsB*, *fhs*, and *metF*, homology arms upstream (FFO-1700/1701, FFO-1854/1855, and FFO-1858/1859) and downstream (FFO-1670/1702, FFO-1856/1857, and FFO-1860/1861) of each gene’s stop codon were PCR amplified from *C. difficile* 630 gDNA. The 3×FLAG-tag was PCR amplified (FFO-1698/1699) from pFF-53 and inserted between the homology arms by SOEing PCR (FFO-1670/1700, FFO-1854/1859, and FFO-1858/1861). The resulting inserts were ligated into pJAK112 using the SacI and BamHI restriction sites, generating pFF-430 (*acsB*-3×FLAG), pFF-501 (*fhs*-3×FLAG), and pFF-502 (*metF*-3×FLAG).

For C-terminally 3×FLAG-tagged CDIF630_02189, ~1 kB homology arms flanking the stop codon were PCR-amplified from wild-type gDNA using FFO-2128/2129 and FFO-2130/2147, with the 3×FLAG sequence appended upstream of the stop codon. To incorporate the native XbaI restriction site from the 3′ UTR of CDIF630_02189 in the upstream homology arm, the upstream homology arm PCR product was subjected to a second PCR amplification using FFO-2146/2156. The resulting upstream homology arm was digested with SacI and XbaI and the downstream homology arm with BamHI and XbaI. Fragments of the correct size were excised from an agarose gel and purified. The products were inserted into SacI- and BamHI-digested, alkaline phosphatase (Invitrogen)-treated pJAK112 by ligation using T4 DNA ligase (Invitrogen), generating pFF-596.

### Plasmid-based sRNA overexpression

#### pFF-176: plasmid-based overexpression of SrsA

For plasmid-based overexpression, the 80-nucleotide region upstream of the *fdxA* TSS was appended to the 5′ end of each sRNA. To achieve this, sRNAs were PCR-amplified from *C. difficile* 630 gDNA, appending an overhang to the *fdxA* promoter (FFO-997/1003). Next, the *fdxA* promoter was amplified from gDNA (FFO-995/996) for SrsA and fused with the sRNA fragment by SOEing PCR. The resulting DNA fragments were inserted into pDSW1728 (65) using the NheI and BamHI restriction sites, generating pFF-176 (SrsA overexpression).

### Reporter constructs

To determine the post-transcriptional effect of SrsA on protein expression, we used an existing fluorescent post-transcriptional reporter system in which the leader and CDS of the mRNA of interest are fused to mCherryOPT (lacking its own start codon) and constitutively transcribed by the moderate *cwp2* promoter (17). This reporter fusion is expressed alone (control) or together with the sRNA of interest, driven by the strong *fdxA* promoter, to determine its post-transcriptional regulatory effect.

#### pFF-322 and pFF-323: construction of post-transcriptional CDIF630_01995 reporter

Since mCherryOPT carries an internal open reading frame, which may uncouple *mCherry* translation from RNA-mediated regulation in the mRNA leader, we introduced a single point mutation in the CDS (G27 to A) that mutates the methionine start codon of the iORF to isoleucine. To this end, the whole mCherry CDS carrying the mutation was amplified (FFO-1407/861) from pFP-441 (kindly provided by Falk Ponath), digested with SacI and BamHI, and introduced into the similarly digested mCherry control vector, yielding (pFF-322). For the mCherry vector with parallel SrsA overexpression, the sRNA was amplified along with the *fdxA* promoter from pFF-176 (FFO-995/998) and inserted using the NheI and XbaI restriction sites of an sRNA overexpression vector, which contains the 5′ UTR and CDS of CDIF630_01995 fused to mCherry. Subsequently, mCherryOPT was replaced with mCherry lacking the iORF by using the SacI and BamHI restriction sites, resulting in pFF-323.

#### pFF-332, pFF-333, pFF-348, pFF-349, pFF-350, and pFF-351: construction of post-transcriptional *fhs* reporter

For testing SrsA-mediated post-transcriptional control of *fhs*, the 392 nucleotides surrounding the *fhs* translation initiation region (comprising the 5′ UTR and the 26 amino acids of the CDS) were PCR-amplified from *C. difficile* 630 gDNA (FFO-1451/1452) and introduced into the control vector (pFF-322) using the XhoI/SacI restriction sites. This resulted in an *fhs*-mCherry control vector driven by the *cwp2* promoter (pFF-332).

For co-expression of SrsA and *fhs*, the *fhs* DNA fragment was introduced into the vector overexpressing the sRNA upstream of the mCherry expression vector (pFF-323) by using the XhoI and SacI restriction sites, generating pFF-333.

To interrogate seed-region specificity, point mutations were introduced into SrsA (“Mt”: C14 to G, G21 to C relative to the TSS) by PCR using *C. difficile* 630 gDNA (FFO-995/1468 + FFO-998/1467) and SOEing PCR (FFO-995/998), and the product was recloned into pFF-333 at the NheI/XbaI site to yield pFF-350 (SrsA*-*Mt + *fhs*-mCherry). To restore targeting, a compensatory *fhs^#^* variant (T25 to G and G18 to C relative to the start codon) was created by PCR using *C. difficile* 630 gDNA (FFO-999/1466 + FFO-998/1465), followed by SOEing PCR (FFO-998/999). The resulting product was then cloned into pFF-332, yielding pFF-348 (*fhs^#^*-mCherry alone); into pFF-333, yielding pFF-349 (wild-type SrsA + *fhs^#^-mCherry*); or into pFF-350, yielding pFF-351 (SrsA-Mt + *fhs^#^-mCherry*), using the XhoI/SacI restriction sites.

#### pFF-330 and pFF-331: construction of post-transcriptional *metF* reporter

To test SrsA-mediated post-transcriptional regulation of *metF,* an insert was created by amplifying the 35 nucleotides upstream of the start codon and the first 54 nucleotides of the CDS from gDNA (FFO-1449/1450). For the control vector expressing the *metF*-mCherry fusion from the *cwp2* promoter, the fragment was inserted using the XhoI and SacI restriction sites of pFF-332, resulting in pFF-331. For the additional constitutive SrsA expression (“WT”) from the *fdxA* promoter, the same restriction sites in pFF-333 were used, producing pFF-330.

SrsA carrying the “Mt” mutation was amplified from pFF-351 using FFO-572/998. The SrsA “M4” mutation was generated by SOEing PCR on the PCR products of FFO-1816/1636 and FFO-995/1817 with pFF-330 as template as input. These fragments were digested with NheI and XbaI and ligated into similarly digested pFF-330, creating FF-592 for the “SrsA-Mt + *metF-mCherry*” reporter and pFF-485 for the “SrsA-M4 + *metF-mCherry*” reporter.

#### pFF-334 and pFF-335: construction of post-transcriptional *lpdA* reporter

The 200 nucleotides upstream and the 14 first codons of the *lpdA* CDS were PCR-amplified from gDNA (FFO-1453/1454), digested with XhoI and SacI, and inserted into similarly digested pFF-332, yielding the reporter control plasmid pFF-334, expressing the *lpdA*-mCherry fusion under the control of the *cwp2* promoter. Insertion of the *lpdA* fragment into similarly digested pFF-333 resulted in the reporter plasmid with parallel SrsA overexpression from the *fdxA* promoter, pFF-335.

#### pFF-406, pFF-407, pFF-414, pFF-415, pFF-529, pFF-530, pFF-532, and pFF-535: construction of anhydrotetracycline-inducible reporter of CDIF630_01677 (CD1511.1) and CDIF630_02189 (CD1980.2)

To evaluate SrsA-mediated post-transcriptional regulation of CDIF630_02189, the gene reaching from the TSS to the stop codon was amplified from gDNA (FFO-1595/1596) and inserted using the restriction sites XhoI and SacI in pFF-332, yielding pFF-406, which expressed CDIF630_02189-mCherry fusion controlled by the *cwp2* promoter.

For the plasmid overexpressing SrsA in addition to CDIF630_02189-mCherry, the PCR product was also inserted into pFF-333 utilizing the same restriction sites, yielding pFF-407. Because the weak fluorescence signal of the reporters did not allow a reliable readout in *C. difficile*, we replaced the moderate *cwp2* promoter with the anhydrotetracycline-inducible *tetR-P_tet_* system.

To this end, the *tetR-P_Tet_* cassette was amplified from pFF-36 (FFO-1614/1616), which also inserted a KpnI restriction site downstream of NheI. As vector backbone, pFF-406 was PCR-linearized (FFO-1595/1480) removing the *cwp2* promoter region, and the *tetR-P_tet_* cassette insert was introduced by Gibson assembly, yielding the CDIF630_02189-mCherry control reporter driven by the inducible *tetR-P_tet_* system (pFF-414). To co-express SrsA constitutively from the same plasmid, SrsA, including the *fdxA* promoter and the *slpA* terminator, was amplified from pFF-333 (FFO-995/1636), and the NheI and KpnI restriction sites were used to introduce the insert into pFF-414. In the resulting construct (pFF-415), CDIF630_02189-mCherry is transcriptionally controlled by the *tetR-P_tet_* system, while SrsA is overexpressed from the *fdxA* promoter.

To investigate the effect of SrsA on CDIF630_01677 (CD1511.1), the 5′ UTR and CDS were amplified from gDNA (FFO-1591/1592) and introduced using the XhoI and SacI restriction sites in pFF-414 and pFF-415, yielding pFF-529, which expresses the CDIF630_01677-mCherry fusion under the control of the *tetR*-P_tet_ system, and pFF-530, which co-expresses SrsA from the *fdxA* promoter.

Specificity of base pairing was examined by generating seed region mutants of SrsA.

“M4” seed mutations (G45 and 46 to C) were introduced into SrsA by PCR on pFF-176 (FFO-1816/1636 and FFO-995/1817) and SOEing PCR (FFO-995/1816). NheI and KpnI restriction sites were used for insertion into pFF-530, generating CDIF630_01677-mCherry under the control of the *tetR-P_tet_* system together with the “M4” mutant of SrsA (pFF-532), or into pFF-415 for the same construct with CDIF630_02189-mCherry (pFF-535).

### Transformation and plasmid propagation in *E. coli*

Ligated plasmids were transformed to competent *E. coli* TopF by heat shock (1 min, 42°C), followed by brief incubation on ice and recovery in LB broth for 1–4 h. After plating on LB agar (20 µg/mL chloramphenicol), positive transformants were identified by colony PCR using Taq polymerase (Biozym). Plasmids were purified from overnight cultures in LB broth (20 µg/mL chloramphenicol) using the NucleoSpin Plasmid Purification Kit (Macherey & Nagel), according to the manufacturer’s instructions. Construct integrity was verified by Sanger sequencing.

For transformations of the donor strain *E. coli* CA434 carrying the conjugative plasmid HB101, 1–2 µL plasmid was added to 80 µL of competent CA434 cells in an ice-cooled electroporation cuvette, followed by a 1.8 kV and 200 Ω electric pulse for 4–5 ms.

After transformations, the bacteria were recovered in LB broth at 37°C with shaking for at least 1 h before an aliquot was plated on LB agar containing 20 µg/mL chloramphenicol for selection.

### Conjugation of plasmids to *C. difficile* 630

To conjugate the plasmid to *C. difficile* 630, 1 mL of an overnight culture of plasmid-harboring *E. coli* CA434 was centrifuged at 5,000 × *g* at RT for 2 min. The pellet was transferred to the Coy anaerobic chamber, suspended in 200 µL of an overnight culture of the respective *C. difficile* strain, and spotted onto BHI agar. After 8 h of incubation, bacteria were collected using 700 µL BHI broth, and 100 µL was streaked out on a BHI agar plate containing 250 µg/mL cycloserine, 15 µg/mL thiamphenicol, and 8 µg/mL cefoxitin.

### Chromosomal manipulation of *C. difficile* 630

For chromosomal manipulations, a previously described two-step allelic chromosomal exchange approach was applied, in which the *C. difficile*-incompatible origin of replication of the vector promotes chromosomal integration, and the second recombination event depends on the *codA* allele, which is lethal in the presence of 5′-fluorocytosine (18, 63).

In brief, plasmids were conjugated to *C. difficile* from *E. coli* CA434, and emerging colonies were restreaked twice to isolate fast-growing clones, which integrated the plasmid into the chromosome. Integration was verified by PCR using one primer that binds to the vector and another that anneals to the chromosomal region flanking the insertion site. Positive clones were streaked on BHI agar and grown for ~ 72 h to allow sporadic second-crossover events, which were identified by harvesting all colonies with PBS and spreading serial dilutions of the mixture on *C. difficile* minimal medium (CDMM) agar plates containing 50 µg/mL 5′-fluorocytosine for counterselection.

Emerging colonies were streaked on BHI and BHI-thiamphenicol agar, and clones growing only on BHI were screened using primers annealing adjacent to the chromosomal region encoding the homology arms. Positive clones were verified by Sanger sequencing of the PCR product.

### Growth curves

Overnight and day cultures were grown in autoclaved TY medium (30 g/L tryptone-peptone [Roth], 20 g/L yeast extract [Roth]). For conditioned autoclaved TY broth, NaCl was added, or the pH was adjusted using 37% HCl. Media were subsequently sterile filtered.

Overnight cultures were inoculated from single colonies into a volume of 5 mL and subsequently diluted to an OD of 0.05 in fresh medium. During exponential growth phase, cultures were diluted into conditioned TY medium to an OD of 0.05. A 200 µL aliquot was transferred to transparent 96-well plates (Nunclon) and incubated statically in a Synergy H1 microplate reader at 37°C with orbital shaking for 10 s prior to OD_600_ measurement every 20 min.

### Plate-based stress assays

For plate-based stress conditions, autoclaved TY agar was cooled to ~45°C, supplemented with a sterile-filtered stressor stock solution, and poured into square Petri dishes. Plates were prepared on the day prior to the assay and deoxygenated overnight in the anaerobic chamber. Bacterial overnight cultures in TY broth were inoculated from plates and used to inoculate main cultures in TY to an OD of 0.05. Main cultures were 10× serially diluted in fresh TY broth and subsequently spotted onto agar plates in 10 µL spots. For diamide stress, spot volume was reduced to 5 µL. Plates were incubated for 24 h, or until CFU spots appeared, at 37°C under anaerobic conditions, or at 1% O_2_ in a hypoxic incubator, where indicated.

### RNA extraction upon σ^B^ overexpression from anhydrotetracycline-inducible promoter

*C. difficile* 630Δ*erm*Δ*sigB* cultures carrying a control plasmid expressing either codon-optimized split luciferase (pAP24) or the *sigB* gene (pWKS17250) from an anhydrotetracycline-inducible promoter (pAP24) were inoculated from exponentially growing day cultures to an OD_600_ of 0.05 in TY broth (15 µg/mL thiamphenicol). At an OD of 0.5, expression from the plasmid was induced with anhydrotetracycline dissolved in ethanol at a final concentration of 100 ng/mL, or with ethanol alone as control. Thirty minutes after induction, 4 mL of culture was harvested and extracted by applying the hot phenol RNA extraction protocol.

### sRNA induction upon stress exposure

*C. difficile* cultures were inoculated from exponentially growing day cultures to an OD_600_ of 0.05. The different stress conditions were prepared as 5× concentrated stocks in anaerobic TY medium. Upon reaching an OD of 0.5, 4 mL of culture was added to 1 mL of each stress condition stock. For exposure to 1% oxygen, 5 mL of culture was poured into a Petri dish and incubated in a hypoxic incubator (1% O_2_, 5% CO_2_) or in an aerobic incubator to expose the culture to ambient oxygen levels. For incubation under different pH conditions, bacteria were centrifuged (8,000 × *g*, 5 min at RT), and the pellet was suspended in anaerobic, pH-adjusted TY broth. After 30 min of treatment, cultures were harvested for RNA isolation with the hot phenol extraction method.

### Hot phenol extraction of total RNA and DNase I digestion

Total RNA was isolated using the hot phenol method. Samples were collected by adding ice-cold Stop Mix (95% EtOH, 5% acidic phenol) corresponding to 20% of the culture volume, and the samples were snap-frozen in liquid N_2_. To extract the RNA, samples were thawed on ice and centrifuged at 4,000 × *g* at 4°C for 20 min. For lysis, pellets were dissolved in 600 µL of 10 mg/mL lysozyme in Tris-EDTA (TE) buffer (pH 8) and incubated at 37°C for 10 min. Following lysozyme treatment, 60 µL of 10% (wt/vol) SDS was incorporated, and the samples were incubated in a water bath at 64°C for 1–2 min after mixing by inversion. Next, 66 µL of 3 M NaOAc, pH 5.2 containing 1 mM EDTA, followed by 750 µL of acidic phenol (Roth), were added. Samples were maintained in a water bath at 64°C for 6 min, and tubes were regularly inverted to mix phases. For phase separation, tubes were centrifuged at 16,000 × *g* at 4°C for 15 min. The upper aqueous layer was transferred into a phase lock gel tube (5PRIME), mixed with an equal volume of chloroform, and centrifuged at 16,000 × *g* at 15°C for 12 min. The recovered aqueous phase was combined with 2 volumes of a 30:1 EtOH-to-3 M NaOAc (pH 6.5) mixture in a new tube and inverted several times. RNA was precipitated at −20°C overnight. After precipitation, samples were pelleted at 16,000 × *g* at 4°C for 30 min and washed twice with 500 µL of ice-cold 75% ethanol, with centrifugation for 10 min at 4°C between washes. The liquid was completely removed, and after air-drying the pellet for ~5 min at RT, 50 µL of RNase-free water was added for elution, which was promoted by shaking at 1,000 × rpm at 65°C for 10 min. Concentration and purity were measured using a Nanodrop 2000 spectrophotometer (Thermo Fisher).

For genomic DNA removal, 5 µg of total RNA was treated with 5 U DNase I and 10 U RNase inhibitor (Takara) for 1 h at 37°C. The sample volume was adjusted to 100 µL with DEPC-treated ddH_2_O and mixed with an equivalent volume of P:C:I for RNA (Roth) in a phase lock gel tube. After phase separation at 16,000 × *g* at 4°C for 15 min, the aqueous layer was transferred to a new tube. A 2.5-volume amount of 30:1 EtOH-to-NaOAc (pH 6.5) was added, and the samples were precipitated overnight at −20°C. RNA was pelleted at 16,000 × *g* at 4°C for 30 min. The pellet was washed twice with 200 µL of 75% EtOH, with centrifugation at 16,000 × *g* at 4°C for 10 min between washes. The supernatant was removed, and the pellet was air-dried for 5 min at RT. The RNA was then eluted in 20 µL DEPC-treated water by shaking at 1,000 × rpm at 65°C for 5 min.

### Reverse transcription and qPCR

Per sample, 1 µg of DNase I-digested RNA was converted to cDNA using the M-MLV Reverse Transcriptase Kit (Invitrogen) with random hexamer primers (Invitrogen), following the manufacturer’s instructions.

Per qPCR, 1 µL of cDNA diluted 20-fold in DEPC-treated water was used together with 2× Takyon NO ROX SYBR Master Mix blue TTP (Eurogentec) and 20 nM primers in a volume of 10 µL. For each reaction, at least two technical replicates were performed per biological replicate. The qPCR was carried out using a QuantStudio 5 Real-Time PCR cycler with the following conditions: 3 min denaturation and polymerase activation at 95°C, 40 cycles of denaturation at 95°C for 10 s and 60 s of annealing and extension time at 60°C, followed by a denaturation step at 95°C for 15 s with an ensuing melting curve analysis from 55°C to 95°C using “step and hold” with 0.5°C and 10 s of incubation per step.

### Northern blot

For northern blots, equal amounts of RNA (4–7 µg) were suspended in GLII (95% formamide, 18 mM EDTA, 0.025% SDS, 0.02% bromophenol blue, 0.025% xylene cyanol), boiled for 5 min at 95°C, and cooled on ice for 5 min. Samples were loaded onto a 6% polyacrylamide (PAA) gel containing 7 M urea and separated by electrophoresis for 2 h at 300 V. RNA was subsequently transferred to a Hybond *N* + membrane (GE Healthcare) in a wet blotting chamber containing 1× Tris/borate/EDTA (TBE) buffer at 50 V and 4°C for 1 h and fixed to the membrane by UV crosslinking (254 nm, 0.12 J/cm^2^). Membranes were pre-incubated in Roti Hybri-Quick buffer (Roth) at 42°C for 1 h.

To detect the RNA of interest, a 10 pmol DNA oligonucleotide probe complementary to the target was γ-^32^P labeled by incubation with 5 U of T4 polynucleotide kinase (Thermo Fisher Scientific) and 1 µL of [γ-^32^P]ATP (10 µCi/µL) for 30 min at 37°C. The reaction was adjusted to 20 µL with DEPC-treated water, and leftover [γ-^32^P]ATP was removed by a purification step over a G25 Sephadex column (Cytiva). Probes were hybridized to the membranes at 42°C overnight. Excess probe was removed by washing with 5×, 1×, and 0.5× SSC buffer containing 0.1% SDS for 20 min per step. After air-drying, the blot was exposed to a phosphor screen for 1–7 days. Signal was recorded using a Typhoon FLA7000 series biomolecular imager (GE). Band intensities were assessed using ImageJ v1.53k (66). Before probing with a new oligonucleotide, residual probes were stripped from the membrane by incubating the blot with boiling, distilled water containing 0.1% SDS, followed by three brief washes with distilled water.

### Rifampicin assay

Rifampicin assay was performed as described previously (18). Signal was quantified using ImageJ v1.53k. The half-life of SrsA was calculated using GraphPad Prism 9 with the one-phase decay equation.

### *In vitro* transcription

*In vitro* transcription templates were generated by high-fidelity fusion polymerase PCR using *C. difficile* 630 gDNA as a template and primers that annealed a T7 promoter at the 5′ end of the sense strand. The sequence of SrsA was amplified using primers FFO-955/956. The mRNA fragments of *metF* (reaching 100 nucleotides upstream of the start codon and 156 nucleotides into the coding sequence), *fhs* (starting from the TSS and reaching 78 nucleotides into the CDS), *lpda* (starting 100 nucleotides upstream of the translation initiation site and reaching 42 nucleotides into the CDS), and CDIF630_01677 (starting from the transcription start site 71 nucleotides upstream of the start codon and covering the CDS and 60 nucleotides of the 3′ UTR) were amplified using primers FFO-1761/1762, FFO1765/1766, FFO-1767/1768, and FFO-1936/1937, respectively.

*In vitro* transcription was performed using the T7 MEGAscript RT Kit (Thermo Scientific) according to the manufacturer’s instructions; the products were then resolved on 6% PAA gels containing 7 M urea, excised, and isolated by overnight incubation in 750 µL RNA elution buffer (0.1 M NaOAc, 0.1% SDS, 10 mM EDTA) at 1,000 × rpm and 8°C. RNA was purified by adding 750 µL of P:C:I for RNA (Roth), thoroughly mixed, and phases were separated by centrifugation. The aqueous phase was transferred to a new tube, and RNA was precipitated overnight at −20°C by adding 1 mL of 30:1 EtOH:NaOAc (3 M, pH 6.5). RNA was collected by centrifugation at 16,000 × *g* at 4°C for 30 min, and the pellet was washed with 250 µL of ice-cold 75% EtOH. After centrifugation for 10 min at 4°C and 16,000 × *g*, the supernatant was fully removed, and the RNA was air-dried at room temperature, followed by elution in 20 µL of RNase-free water.

### Dephosphorylation and 5′ labeling of RNA

For dephosphorylation, 50 pmol of *in vitro*-transcribed RNA was digested with 25 U of calf intestinal alkaline phosphatase (NEB) in a total volume of 50 µL at 37°C for 1 h. Subsequently, the RNA was purified by phase separation with P:C:I, isolated from the aqueous phase by EtOH:NaOAc precipitation, pelleted, washed with 75% EtOH as described above, and eluted in 20 µL. For radioactive 5′-end labeling, 20 pmol of dephosphorylated RNA was mixed with 10 U T4 polynucleotide kinase (Thermo Scientific) and 2 µL of [γ-32P]ATP (10 µCi/µL) in a total volume of 20 µL of 1× PNK Buffer A (Thermo Scientific). These reactions were incubated for 30 min at 37°C. Remaining free [γ-^32^P]ATP was removed by purification step using a Sephadex G-50 column (Cytiva), and the labeled RNA was additionally separated on a denaturing PAA gel. The full-length RNA was excised from the gel and isolated by overnight incubation in RNA elution buffer, followed by organic phase separation, precipitation of the aqueous phase, and elution as described above.

### Lead(II)-based structure probing

A 0.4 pmol sample of γ-^32^P 5′ end-labeled RNA was allowed to fold for 10 min at 37°C in 1× structure buffer (10 mM Tris-HCl [pH 7.0], 0.1 M KCl, 10 mM MgCl_2_) containing 1 µg of yeast RNA (Invitrogen) per reaction. Next, unlabeled interactor RNA in 1× structure buffer or only structure buffer as a control was added to a final volume of 10 µL, which was incubated at 37°C for another 15 min. To induce cleavage of single-stranded RNA, 2 µL lead(II) acetate was added to a final concentration of 25 nM. The reaction was stopped after 90 s by adding 12 µL GLII, and the samples were transferred to ice.

Per control and ladder, 2 pmol of 5′ end-labeled RNA was denatured at 95°C for 2 mins. As an untreated control, RNA was added to 8 µL of water and boiled at 95°C for 2 min. For the nucleotide ladder, the labeled RNA was added to 9 µL alkaline hydrolysis buffer (Invitrogen) and kept at 95°C for 5 min. For the RNase T1 ladder, denatured RNA was added to 9 µL of 1× sequencing buffer containing 0.1 U of RNase T1 (Invitrogen), followed by incubation at 37°C for 5 min. Reactions were stopped by adding 12 µL of GLII. RNA was denatured for 2 min at 95°C, and half the volume of each reaction was separated on 8% PAA/7 M urea or 10% PAA/7 M urea sequencing gels. Gels were transferred onto Whatman paper and dried in a gel drier 583 (BioRad) for 2 h at 80°C. Signal was collected by exposure to a phosphor screen, which was read out on a Typhoon FLA7000 series biomolecular imager (GE).

### Electrophoretic mobility shift assays (EMSA)

To investigate RNA-RNA interactions with electrophoretic mobility shift assays, one RNA was radioactively labeled and incubated with increasing amounts of RNA interactor. A 0.04 pmol sample of radio-labeled RNA was used per reaction. The labeled RNA stock was denatured at 95°C for 1 min and chilled on ice for 5 min. Then, 1× structure buffer was added, and the RNA was folded at 37°C for 10 min. Subsequently, yeast RNA (Ambion), equivalent to 1 µg per reaction, was added, and the labeled RNA was aliquoted to tubes containing increasing concentrations of unlabeled RNA or recombinant *C. difficile* 630 Hfq (18). Reactions were incubated at 37°C for 15 min, 5× RNA native loading buffer was added, and complexes were separated on native 6% PAA gels at 4°C in 0.5% TBE at a constant current of 40 mA for 3–4 h. Gels were dried onto Whatman paper and exposed to a phosphor screen for 1–2 days. Signals were detected on a Typhoon FLA7000 series biomolecular imager (GE) and quantified with ImageJ v1.53k (66).

### Western blot

Bacterial cultures (equal to 1 or 2 OD_600_ units) were collected in 2 mL Eppendorf tubes and transferred to ice, then centrifuged for 10 min at 4°C and 10,000 × *g*. After removal of supernatant, the centrifugation step was repeated for 5 min, and the remaining liquid was removed. Pellets were stored at −20°C. For lysis, samples were dissolved in 60 µL of 1× protein loading buffer (62.5 mM Tris-HCl, pH 6.8; 100 mM DTT; 10% glycerol [vol/vol]; 2% SDS [vol/vol]; 0.01% bromophenol blue) per OD_600_ unit and boiled at 95°C for 10 min. Per sample, 0.5 OD_600_ units were separated on 10% or 15% SDS-PAGE gels and blotted onto a 0.2 µm nitrocellulose membrane (Cytiva) by semi-dry transfer. Whole-protein staining with Ponceau S (Sigma-Aldrich) served as a loading control. Membranes were blocked with 5% skim milk in 1× TBST for 1 h at room temperature, then incubated with primary mouse monoclonal anti-FLAG antibody (Sigma #F1804, diluted 1:2,000 in 5% milk, 1× TBST) at 4°C overnight with shaking. After three washes with 1× TBST for 10 min each, secondary HRP-coupled anti-mouse antibody (Thermo Fisher #31430, 1:10,000 in 5% skim milk, 1× TBST) was added and incubated for 1 h at room temperature with shaking. After three washes with 1× TBST, Amersham ECL detection reagent (Cytiva) was applied according to the manufacturer’s instructions, and chemiluminescence was detected using an Amersham ImageQuant CCD imager (Cytiva).

### Sporulation assays

Sporulation assays were performed using 70:30 sporulation broth (67). Briefly, overnight TY cultures were used to inoculate day cultures in BHIS containing 0.5% fructose and 0.1% taurocholate to an OD_600_ of 0.05. Upon reaching the mid-exponential growth phase, 70:30 broth was inoculated to an OD_600_ of 0.01. At the end of the transition phase (~8–10 h after inoculation), the total CFUs were assessed from untreated cultures by serial dilution in BHIS (BHI + 5 g/L yeast extract) broth and spotting on BHIS agar (10 µL spots in technical triplicate). In addition, a 30-min ethanol-treated aliquot (48% [vol/vol] final concentration) was serially diluted in PBS and spotted onto BHIS agar with 0.1% taurocholate to check for spore carryover. This time point is referred to as 0 h. At subsequent time points, vegetative CFUs were assessed by serial dilution and spotting on BHIS agar. Spores were assessed from the same cultures by serial dilution and spotting onto BHIS plates containing 0.1% taurocholate, following ethanol treatment as described above.

### Preparation and fluorescence quantification of reporter strains

Single colonies were picked from BHI agar plates containing 15 µg/mL thiamphenicol for overnight cultures in TY broth (15 µg/mL thiamphenicol). The next day, main cultures were inoculated to an OD of 0.05. Reporter strains expressing the mCherry fusion under the control of the *cwp2* promoter were harvested during the late exponential growth phase (OD ~ 0.8).

Strains with anhydrotetracycline-inducible reporter were grown to mid-exponential phase (OD 0.5), induced with 10 ng/mL ATc, and grown for an additional 3 h.

For collection, 200 µL of culture was transferred to a 96-well plate and centrifuged at 4,500 × *g* and 4°C for 10 min. Supernatant was carefully aspirated using a multichannel pipette, and bacteria were resuspended in 150 µL 4% PFA and fixed at RT in the dark for 15 min. After three washes with 200 µL PBS, with centrifugation steps as described above, the final cell pellet was resuspended in 50 µL PBS containing 1 µg/mL DAPI and stored overnight in the dark at 4°C to allow fluorophore maturation under aerobic conditions. Before analysis, cells were washed, centrifuged, and resuspended in 200 µL of PBS and subsequently diluted to an appropriate concentration. Flow cytometry was conducted on a Novocyte Quanteon flow cytometer (Agilent), recording 100,000 DAPI-positive events per sample. DAPI-positive cells were identified using a 405 nm laser and the “Pacific-Blue” filter (detecting 445 ± 25 nm) with an FSC-H (>5,000 AU) cutoff to discriminate cells from debris events. Red fluorescence was measured using a 561 nm laser with a “PE-Texas Red” bandwidth filter (detecting 610 ± 10 nm). The mean fluorescence intensity was calculated from the DAPI-positive gated population.

### *In silico* structure prediction of SrsA and base-pairing interactions

RNA secondary structures were predicted using the RNA-fold web server (29) and were visualized using the Varna applet (68).

Base-pairing predictions were performed with IntaRNA (69), allowing G-U base pairing at helix ends and otherwise default conditions.

### SrsA motif detection in RIL-Seq

Sequences of Hfq RIL-seq interactions comprising more than 30 unique chimeric reads, including 50 nucleotides downstream, were fetched and entered into MEME. The minimum motif width was set to 7, the maximum motif width to 15, and the minimum site number per motif was set to 30. Other parameters were left at default settings.

### RIL-seq data visualization

Circos plots of the RIL-seq data were generated as previously described (17). Data were obtained using the script plot_circos_plot.py from the RIL-seq computational pipeline (https://doi.org/10.48550/arXiv.1303.3997). Circos plots were generated with Circos. To avoid overloading the plots, only a fraction of the interactions is shown, as specified in the figure description.

### Visualization of SrsA interaction regions along target coding sequences

The positions of SrsA RIL-seq interaction boundaries (start and interaction end + 50 nucleotides downstream) and the coding sequence (CDS) coordinates of target genes were obtained from the supplemental data in Fuchs et al. (17). The distance of each interaction boundary from the CDS end was calculated and normalized to the CDS length. Normalized positions were transformed as 1 – *d*, where *d* is the normalized distance from the CDS end, resulting in a coordinate system in which 0 corresponds to the beginning of the start codon and 1 to the end of the stop codon. Interaction regions were visualized as horizontal intervals along normalized CDS coordinates, with each row representing one gene.

### Preparation of spore stocks and inoculum

Spore stocks were prepared as described previously (70). Single colonies from vegetatively growing wild-type *C. difficile* 630 (WT, FFS-1) and *C. difficile* 630 Δ*acsB* (OE, FFS-1287) were picked from BHI agar, streaked onto 70:30 sporulation agar plates, and incubated at 37°C for 5 days. Subsequently, cells were collected by adding 1.5 mL double-distilled sterile water per agar plate and scraping with an inoculation loop. The suspension was transferred to microcentrifuge tubes and kept under aerobic conditions at 4°C for at least 5 days to kill vegetative cells. To purify the spores, 1.5 mL of spore suspension was carefully layered on top of 10 mL of 50% (wt/vol) sucrose solution in 15 mL conical tubes. Tubes were centrifuged for 4,000 × *g* for 20 min at 4°C, supernatant was removed, and the spore pellet was resuspended in 500 µL of sterile, double-distilled water and transferred to a microcentrifuge tube. The pellet was washed five times with intermediate centrifuging steps at 14,000 × *g* for 5 min at RT. Spores were suspended in 1 mL of sterile, double-distilled water and stored in a glass vial. Purity was assessed under a phase-contrast microscope.

To determine the concentration of each stock, *C. difficile* 630 WT and OE mutant spore stocks were first heated at 65°C for 20 min to kill any remaining vegetative cells. Following this heat treatment, each spore stock was added to a 96-well plate and serially diluted 1:10 in sterile PBS (Gibco #10010023). Each dilution was plated in duplicate on taurocholate cycloserine cefoxitin fructose agar (TCCFA). The resulting number of colony-forming units per milliliter of spore stock plated (CFU/mL) was enumerated after 24 h of anaerobic incubation at 37°C.

To prepare *C. difficile* spores for oral gavage, spore stocks were heat-treated as described above. Each spore stock was then added to sterile PBS to create an inoculum with a dose of 10^4^ spores in 100 μL per mouse (42). To confirm the spore concentration, residual inoculum was diluted, plated, incubated, and enumerated as described above.

### Mouse models of *C. difficile* infection

Four-week-old C57BL/6 male mice ordered from The Jackson Laboratory were given 1 week to acclimate, with a bedding swap performed between cages to equilibrate microbiota prior to experimentation. To induce susceptibility to *C. difficile* infection, 0.5 g/L of cefoperazone (Sigma-Aldrich #C4292-5G) was administered in the drinking water *ad libitum* for 5 days. Mice were given a 2-day recovery period before challenge with 10^4^ spores of either *C. difficile* 630 WT (FFS-1) or *the* Δ*srsA pyrE::PslpA-srsA* (OE) mutant (42). All mice were culture-negative for *C. difficile* prior to infection. Mice were monitored daily for weight loss and euthanized if weight loss exceeded 20% of initial mouse body weight. To quantify disease severity at select time points, weight loss, stool quality, and behavior were graded on a scale of 0 to 4 to create a composite clinical sickness score (CSS). Any mouse whose weight loss exceeded 20% of their original body weight was euthanized.

In the relapse infection model, a subset of *C. difficile* 630 WT- and OE-infected mice were given 0.4 g/L of vancomycin (NDC 0143-9162-10) in the drinking water *ad libitum* for 5 days, starting on day 4 post-infection (44). Mice were then returned to regular drinking water and monitored daily for clinical signs of relapse infection, such as weight loss, diarrhea, and lethargy.

### *C. difficile* burden and toxin titer enumeration from stool

To determine the *C. difficile* burden in the stool, fecal pellets collected at select time points were weighed, homogenized in 1 mL of sterile PBS, and added to a 96-well plate to undergo serial 1:10 dilutions in sterile PBS. Each dilution was plated on TCCFA and incubated under anaerobic conditions at 37°C for 24 h. Following incubation, the number of colony-forming units at the lowest countable dilution per amount of sample plated (CFU/mL) was enumerated and normalized to stool weight (in grams).

To quantify the spore burden in the stool, 100 µL aliquots of each stool homogenate were heat treated at 65°C for 20 min to kill any vegetative cells. These stool homogenates were then diluted, plated, incubated, and enumerated as described above.

To prepare the stool samples for the cytotoxicity assay, homogenates were first pelleted by centrifugation at 4,000 rpm for 5 min. The resulting supernatants were then filtered through a 0.2 µm filter during a 5-min spin at 700 rpm. The filtered supernatants were then serially diluted 1:10 in sterile PBS in a 96-well plate. These dilutions were then added to Vero cell monolayers (ATCC CCL-81) for overnight incubation at 37°C in 5% CO_2_ (41, 42). The reciprocal value of the highest dilution that rounded 100% of Vero cells was normalized to stool weight to quantify the toxin titer per gram of stool.

## SUPPLEMENTAL MATERIAL

Figure S1 mbio.01923-26-s0001.tif Sequence conservation and σ^B^-dependent induction of SrsA.

Figure S2 mbio.01923-26-s0002.tif Hfq dependence of SrsA.

Figure S3 mbio.01923-26-s0003.tif SrsA regulates a family of highly conserved small proteins.

Figure S4 mbio.01923-26-s0004.tif SrsA has multiple target sites in the Wood-Ljungdahl operon.

Figure S5 mbio.01923-26-s0005.tif Quantification of protein levels of 3×FLAG-tagged Fhs, MetF, and AcsB.

Figure S6 mbio.01923-26-s0006.tif SrsA enhances pathogenicity in a mouse model of relapse *C. difficile* infection.

Legends mbio.01923-26-s0007.docx Supplemental figure legends.

Table S1 mbio.01923-26-s0008.xlsx RIL-seq RNA pairs identified in unified data sets of *C. difficile hfq*::3×FLAG strains generated by Fuchs et al.

Table S2 mbio.01923-26-s0009.xlsx Locus tags of paralogous protein family.

Table S3 mbio.01923-26-s0010.xlsx Plasmids, oligonucleotides, and strains.

ASM does not own the copyrights to Supplemental Material that may be linked to, or accessed through, an article. The authors have granted ASM a non-exclusive, world-wide license to publish the Supplemental Material files. Please contact the corresponding author directly for reuse.
