# Supplementary material for: A σB-dependent sRNA provides a repressor function to the general stress response in Clostridioides difficile
Source: mBio. 2026 Oct 26;17(11):e01923-26. doi: 10.1128/mbio.01923-26 (PMC13753753; doi:10.1128/mbio.01923-26)
Supplement: Supplementary file 1 [file mbio.01923-26-s0007.docx]

**Supplementary figure descriptions**

**Figure S1. Sequence conservation and σ^B^-dependent induction of SrsA.**

**A** SrsA sequence and promoter alignment across representative strains from the 5 genomic clades of *C. difficile* (24) created with MultAlin (55)using default settings with the DNA symbol comparison table. TSS is indicated with a bent arrow. The σ^B^ consensus motif is shown below, and conserved -35 and -10 promoter regions are highlighted in boxes. The σ^B^ consensus motif was determined by fetching the promoter sequences (18)(18) of all genes more than twofold upregulated during σ^B^ pulse expression (23)(23), and searching for a consensus motif with MEME(29). The resulting motif highly resembles the previously reported motif (3)(3).

**B** SrsA expression after 30 minutes of exposure to a panel of stress conditions. One representative northern blot of biological triplicates is shown. 5S rRNA serves as a loading control.

**C** Growth curve of cultures used for RNA isolation and Northern blot in Figure 1**D**. Mean of OD_600_ ± SD of biological triplicates is shown.

**D** Serial dilutions of WT, Δ*srsA,* and Δ*srsA pyrE::P_slpA_-srsA* (constitutive chromosomal expression, OE) spotted on TY agar Petri dishes containing indicated stressors. For 1% oxygen exposure, dilutions were spotted onto TY agar and incubated in a hypoxic incubator. One representative of four biological replicates is shown.

**E** Quantification of survival after stress exposure. Fold change to TY ctrl was calculated by dividing CFU/mL under stress by CFU/mL in the ctrl condition (TY agar). As no single colonies grew at 1% oxygen and pH 5, the last dilution exhibiting visible growth was used for survival calculations. The mean ± SD of four biological replicates is shown. Significance of differences between strains under the same stress condition was determined by two-way ANOVA with Tukey’s multiple comparisons test. Significance is displayed as ** p < 0.01.

**Figure S2. Hfq-dependence of SrsA**

**A** EMSAs performed with 4 nM of radioactively 5’ phosphate-labeled SrsA incubated with increasing concentrations of purified *C. difficile* 630 Hfq. One representative of three replicates is shown.

**B** Quantification of EMSAs performed with 5’ phosphate-labeled SrsA incubated with Hfq. Shown is the mean band intensity ± SD from three independent replicates.

**C** Overlay of the SrsA interactome determined by RIL-seq with the σ^B^ regulon determined by induced σ^B^ overexpression (23)(23). Multiple RIL-seq interactions within the same gene were counted as one. Individual genes within the intersection are shown below the diagram. The promoter analysis for the σ^B^ recognition motif of all genes in the intersection is shown in Figure S2D.

**D** Promoter analysis of transcripts that form chimeras with SrsA in RIL-seq and are also differentially regulated during σ^B^ pulse expression. High-confidence SigB promoters are highlighted with a red dashed box. If two TSSs are annotated, the one containing a σ^B^ promoter is shown. Secondary TSSs are annotated as [sTSS]. The first transcribed nucleotide is indicated in bold, and the σ^B^ motif is highlighted in red in the consensus sequence.

**E** Global overview of SrsA interactions within the target mRNA coding sequences captured by Hfq RIL-seq, sorted by chimeric read abundance. The interaction region (from the first chimeric read to 50 nucleotides downstream of the last chimeric read) was mapped to the corresponding mRNA coding sequence (CDS boundaries marked by pink lines) and surrounding region. Each row, representing one interaction, was normalized to the size of the corresponding CDS. Chimeric reads mapping to antisense transcripts, intergenic or 3’ UTR-derived ncRNAs are excluded. Genes overlapping with the σ^B^ regulon are marked in red.

**Figure S3. SrsA regulates a family of highly conserved small proteins**

**A** Amino-acid sequence alignment of the family of eight small protein paralogs from *C. difficile* 630. Sequences are colored according to the BLOSUM62 scheme and sorted according to the BLOSUM62 Neighbor-Joining Tree generated with Jalview (64)(56).

**B** Pairwise amino-acid sequence identity between the eight small all 8 protein paralogs. Percentage of identical amino acids is indicated for each comparison.

**C** Detection of CDIF630_01677, CDIF630_02189 mRNA and the CDIF630_02189-associated premature transcription termination product of the c-di-GMP I riboswitch (“PTT”) by northern blot. RNA samples were collected during growth in TY at mid-exponential phase (ME, 4 h post-inoculation), early stationary phase (eSP, 8 h post-inoculation), stationary phase (SP, 12 hours post-inoculation), and late stationary phase (LS, 24 h post-inoculation). One representative of three biological replicates is displayed. 5S rRNA served as a loading control. SrsA signal in the constitutive overexpression strain (Δ*srsA* p*srsA*) relative to wild-type carrying an empty vector control (WT-pEV) is shown as the mean ± SD of three biological replicates.

**D** Quantification of northern blot and replicates shown in C. Signals are normalized to the 5S rRNA loading control and displayed as fold change to the wild-type (WT-pEV) at mid-exponential phase. Data are shown as mean ± SD from three biological replicates. Statistical significance was determined by a two-way ANOVA with Tukey’s multiple comparisons test and is indicated as * p < 0.05 and ** p < 0.01.

**E** Western blot detection of chromosomal C-terminally 3xFLAG-tagged CDIF630_02189 protein from cultures grown in 70:30 medium. Strains harbor an empty vector (pEV) control or a plasmid for SrsA overexpression from the constitutive fdxA promoter (p*srsA*). One representative of three biological replicates is displayed. Whole protein staining with Ponceau S served as a loading control.

**F** Quantification of 3xFLAG signal for all biological replicates of the western blot analysis shown in E. The signal was normalized to the Ponceau S loading control in each lane. Data are shown as mean ± SD from three biological replicates. Statistical significance was determined by two-way ANOVA with Sidak’s multiple comparisons test and is indicated as * p < 0.05 and ** p < 0.01.

**Figure S4. SrsA has multiple target sites in the Wood-Ljungdahl operon**

**A-C** EMSAs of in vitro transcribed ^32^P-5’-end-labeled SrsA with increasing concentrations of in vitro transcribed *fhs*, *metF,* and *lpdA* mRNA fragments. The band intensity was quantified from three independent replicates and is depicted below with the dissociation constants.

**D-F** Lead(II) acetate-based structure probing of 0.2 pmol ^32^P-5’-end-labeled (*) SrsA in the absence or presence of *fhs* (**D**), *metF* (**E**), and *lpdA* (**F**) mRNA fragments. Shown is one representative of three replicates. Untreated RNA was loaded as a control (Ctrl); the partially RNase T1-digested RNA was used for a G ladder (T1), and a single-nucleotide ladder was created by alkaline hydrolysis of the RNA (OH). Addition of Lead(II) acetate as a cleavage agent is indicated with ‘Pb(II)’. Ratio of unlabeled RNA interactors to labeled RNA in each lane is indicated below. The predicted secondary structure of SrsA alone is displayed with solid black lines for base-paired regions and dashed gray lines for single-stranded regions. The protected regions upon mRNA incubation are indicated with a red line.

**Figure S5. Quantification of protein levels of 3xFLAG-tagged Fhs, MetF and AcsB**

**A** Quantification of SrsA target mRNA levels by RT-qPCR. Levels are normalized to the 16S rRNA control, and subsequently the fold change to the wild-type during the mid-exponential growth phase was calculated. Data are shown as mean ± SD of biological triplicates. Significance was determined by two-way ANOVA with Tukey’s multiple comparisons test and is indicated as * p < 0.05, ** p < 0.01.

**B** Western blot quantification of protein levels of chromosomally 3xFLAG-tagged Fhs, 3xFLAG-tagged MetF, and 3xFLAG-tagged AcsB in strains carrying an SrsA overexpression plasmid or an empty vector control plasmid. The log_2_ fold change of the SrsA overexpression strain relative to the empty vector control at the same growth phase is shown. Signals were normalized to the Ponceau S loading control. Data are shown as mean ± SD. Significance was determined by two-way ANOVA with Dunnett’s multiple comparisons test and is indicated as * p < 0.05, ** p < 0.01.

Time points in A and B represent mid-exponential (ME, 4 hours), early stationary (eST, 8 hours), stationary (ST, 12 hours), and late stationary phase (LS, 24 h).

**Figure S6. SrsA enhances pathogenicity in a mouse model of relapse *C. difficile* infection**

**A** Schematic of a mouse model of relapse *C. difficile* infection. Cefoperazone (abx) was supplemented in the drinking water *ad libitum* for five days to make mice susceptible to CDI. After a two-day recovery, mice were challenged with 10^4^ spores of *C. difficile* 630 wild-type (WT) or Δ*srsA pyrE::PslpA-srsA* (OE). Following the acute phase of infection, drinking water was supplemented with vancomycin (vanco) from day 4 through 9 post-infection to clear CDI. Mice were then placed back on regular drinking water and monitored for the recurrence of CDI. Mouse weight and clinical sickness score (CSS), as well as *C. difficile* bacterial burden (CFU), toxin titer, and spore burden from stool, were assessed at selected time points (gray triangles).

**B-C** Mouse weight (**B**) and clinical sickness (**C**) were monitored throughout the course of infection.

**D-F** *C. difficile* bacterial burden (CFU) (**D**), spore burden (**E**), and toxin titer (**F**) were quantified in stool at the indicated time points.

Data in **B-F** are represented as the mean ± SD. Statistical significance was determined by two-way ANOVA with multiple Mann-Whitney tests.

**G** Survival curve of acute and relapse mouse infection model by Kaplan-Meier analysis with log-rank test comparison with Holm’s correction.
